# Supplementary figures and images for: Functional analysis of the nonstructural protein NSs of tomato zonate spot virus
Source: PLoS One. 2022 Jan 24;17(1):e0262194. doi: 10.1371/journal.pone.0262194 (PMC8786149; doi:10.1371/journal.pone.0262194)

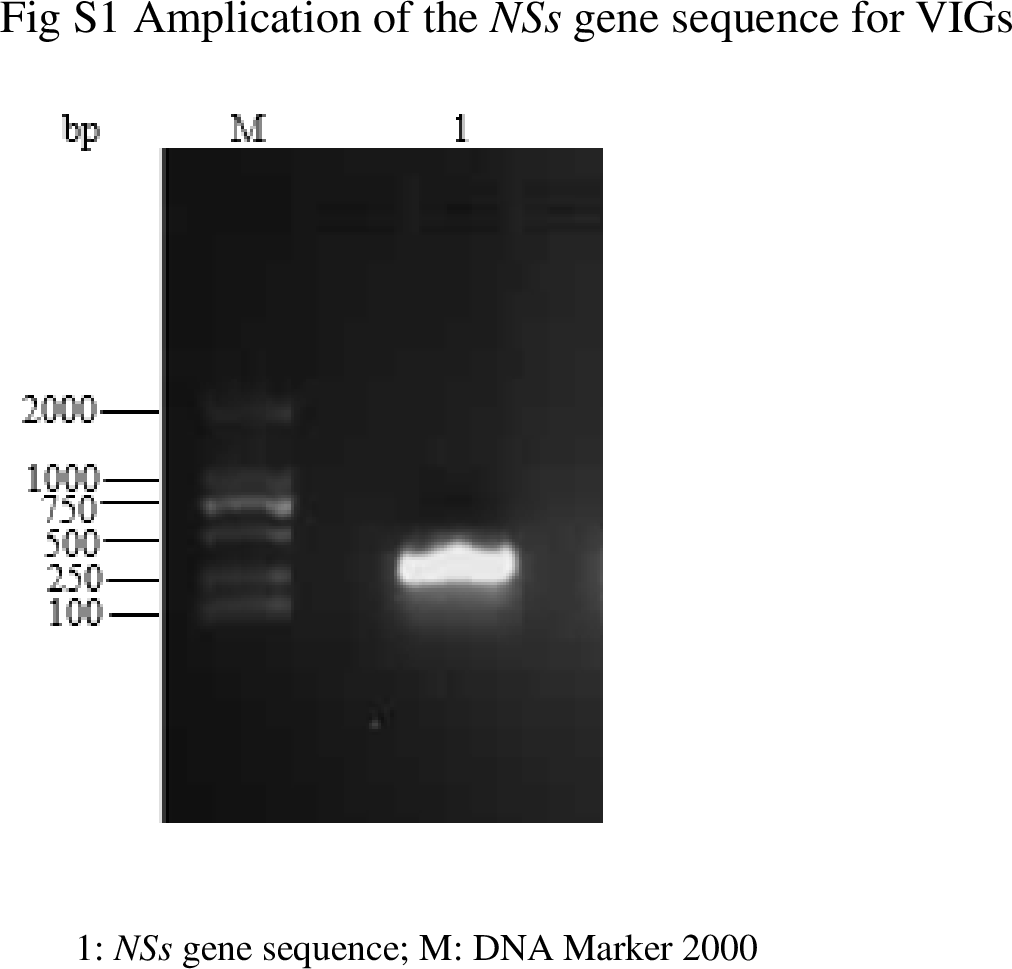

Supplement: S1 Fig — (TIF) [file pone.0262194.s001.tif]
