# Supplementary material for: Functional analysis of the nonstructural protein NSs of tomato zonate spot virus
Source: PLoS One. 2022 Jan 24;17(1):e0262194. doi: 10.1371/journal.pone.0262194 (PMC8786149; doi:10.1371/journal.pone.0262194)
Supplement: S1 Table — The primers were used to construct the VIGS, fluorescence labeling, and RT-qPCR vectors. (DOCX) [file pone.0262194.s002.docx]

Table S1 Special primers

| Primer name | Sequence (5'-3') | Fragment length /bp | Primer function |
| --- | --- | --- | --- |
| NSs-G-F | ACGGGGGACTCTTGACCATGGATGTCTACTGCAAAGATGTCTGCT | 1380 | GFP vector constructed |
| NSs-G-R | CTCGCCCTTGCTCACACTAGTAGCAGTTTGAACCTTTTCCTCAGAT |  |  |
| TZNSSQF | GTGCGAGCCATTCAGATAGC | 123 | RT-qPCR |
| TZNSSQR | ATCTTGCACGCCAGGGAAAG |  |  |
| TZNSSVF | CCCAAGCTTTTGTCTACAGTGCGAGCCATT | 138 | VIGS vector constructed |
| TZNSSVR | CGGGATCCACCTTCATCTTGCACGCCA |  |  |
| Zing-m-F | CTAGTCTAGAATGGCTATGAAAATCAATTTGATCA | 1020 | mCherry vector constructed |
| Zing-m-R | CGCGGATCCAATGACTGGATAAGAAGACAACTTG |  |  |
