# Supplementary material for: Functional analysis of the nonstructural protein NSs of tomato zonate spot virus
Source: PLoS One. 2022 Jan 24;17(1):e0262194. doi: 10.1371/journal.pone.0262194 (PMC8786149; doi:10.1371/journal.pone.0262194)
Supplement: Attachment — Submitted filename: PONE-D-21-26999_R1 WSM - 26 Nov 2021.pdf [file pone.0262194.s004.pdf]

PLOS ONE

Functional analysis of the nonstructural protein NSs of tomato zonate spot virus

--Manuscript Draft--

**Manuscript Number:**	PONE-D-21-26999R1
**Article Type:**	Research Article
**Full Title:**	Functional analysis of the nonstructural protein NSs of tomato zonate spot virus
**Short Title:**	Functional analysis of the nonstructural protein NSs of tomato zonate spot virus
**Corresponding Author:**	Lihua Zhao<br>Institute of biotechnology and genmplasm resource, Yunnan academy of agriculture science<br>kunming, CHINA
**Keywords:**	Tomato zonate spot orthotospovirus (TZSV), RNA silencing suppressor, virus induced gene silenced (VIGS), location
**Abstract:**	Tomato zonate spot virus (TZSV), a member of the genus orthotospovirsus, causes severe damage to vegetables and ornamental crops in southwest China. The NSs protein is an RNA silencing suppressor in various orthotospovirsus like TZSV, but its mechanism and role in virus infection are poorly understood. Here, we observed that an NSs-GFP fusion protein was transiently expressed on the plasma membrane and Golgi bodies in Nicotiana benthamiana plants. The TZSV NSs gene was silenced and infiltrated into N. benthamiana and N. tabacum cv. K326. RT-qPCR and Indirect enzyme-linked immunosorbent assay (ID-ELISA) showed that the transcription and the protein expression of the NSs gene were inhibited by more than 90.00%, and the symptoms on silenced plants were alleviated. We also found that the expression of the Zingipain-2-like gene significantly decreased when the NS s gene was silenced, resulting in co-localization of the NSs-GFP and the Zingipain-2-like-mCherry fusion protein. The findings of this study provide new insights into the mechanism of silencing suppression by NSs, as well as its effect on systemic virus infection, and also support the theory of disease resistance breeding and control and prevention of TZSV in the field.
**Order of Authors:**	Jing-Li1¶
	Si-Chen1 2¶
	Run-Shuang Qiu
	Li-Zhen Zhang
	Yue Chen
	Xue Zheng
	Ting-Ting Li
	Lihua Zhao
	Zhong-Kai Zhang
**Opposed Reviewers:**	
**Response to Reviewers:**	Thank you for considering our work and give us an opportunity. We modified the content following the editor and the reviewers' comments, and outline every change point by point.
**Additional Information:**	
**Question**	**Response**
**Financial Disclosure**<br><br>Enter a financial disclosure statement that describes the sources of funding for the	Zhong-kai Zhang National Natural Science Foundation of China (No. U1802235) Li-hua Zhao National Natural Science Foundation of China(No.31960531), Yunnan Fundamental Research Projects (202101AT070269)

work included in this submission. Review the submission guidelines for detailed requirements. View published research articles from *PLOS ONE* for specific examples.<br><br>This statement is required for submission and **will appear in the published article** if the submission is accepted. Please make sure it is accurate.<br><br>**Unfunded studies**<br>Enter: *The author(s) received no specific funding for this work.*<br><br>**Funded studies**<br>Enter a statement with the following details:<br>• Initials of the authors who received each award<br>• Grant numbers awarded to each author<br>• The full name of each funder<br>• URL of each funder website<br>• Did the sponsors or funders play any role in the study design, data collection and analysis, decision to publish, or preparation of the manuscript?<br>• **NO** - Include this sentence at the end of your statement: *The funders had no role in study design, data collection and analysis, decision to publish, or preparation of the manuscript.*<br>• **YES** - Specify the role(s) played.<br><br>* typeset	
**Competing Interests**<br><br>Use the instructions below to enter a competing interest statement for this submission. On behalf of all authors, disclose any competing interests that could be perceived to bias this work—acknowledging all financial support and any other relevant financial or non-financial competing interests.<br><br>This statement is required for submission and **will appear in the published article** if the submission is accepted. Please make sure it is accurate and that any funding sources listed in your Funding Information	The authors have declared that no competing interests exist.

later in the submission form are also declared in your Financial Disclosure statement.<br><br>View published research articles from *PLOS ONE* for specific examples.<br><br>**NO authors have competing interests**<br><br>Enter: *The authors have declared that no competing interests exist.*<br><br>**Authors with competing interests**<br><br>Enter competing interest details beginning with this statement:<br><br>*I have read the journal's policy and the authors of this manuscript have the following competing interests: [insert competing interests here]*<br><br>* typeset	
**Ethics Statement**<br><br>Enter an ethics statement for this submission. This statement is required if the study involved:<br><br>• Human participants<br>• Human specimens or tissue<br>• Vertebrate animals or cephalopods<br>• Vertebrate embryos or tissues<br>• Field research<br><br>Write "N/A" if the submission does not require an ethics statement.<br><br>General guidance is provided below. Consult the submission guidelines for detailed instructions. **Make sure that all information entered here is included in the Methods section of the manuscript.**	N/A

**Format for specific study types**<br><br>**Human Subject Research (involving human participants and/or tissue)**<br>• Give the name of the institutional review board or ethics committee that approved the study<br>• Include the approval number and/or a statement indicating approval of this research<br>• Indicate the form of consent obtained (written/oral) or the reason that consent was not obtained (e.g. the data were analyzed anonymously)<br><br>**Animal Research (involving vertebrate animals, embryos or tissues)**<br>• Provide the name of the Institutional Animal Care and Use Committee (IACUC) or other relevant ethics board that reviewed the study protocol, and indicate whether they approved this research or granted a formal waiver of ethical approval<br>• Include an approval number if one was obtained<br>• If the study involved *non-human primates*, add *additional details* about animal welfare and steps taken to ameliorate suffering<br>• If anesthesia, euthanasia, or any kind of animal sacrifice is part of the study, include briefly which substances and/or methods were applied<br><br>**Field Research**<br><br>Include the following details if this study involves the collection of plant, animal, or other materials from a natural setting:<br>• Field permit number<br>• Name of the institution or relevant body that granted permission	
**Data Availability**<br><br>Authors are required to make all data underlying the findings described fully available, without restriction, and from the time of publication. PLOS allows rare exceptions to address legal and ethical concerns. See the PLOS Data Policy and FAQ for detailed information.	Yes - all data are fully available without restriction

A Data Availability Statement describing where the data can be found is required at submission. Your answers to this question constitute the Data Availability Statement and **will be published in the article**, if accepted.<br><br>**Important:** Stating 'data available on request from the author' is not sufficient. If your data are only available upon request, select 'No' for the first question and explain your exceptional situation in the text box.<br><br>Do the authors confirm that all data underlying the findings described in their manuscript are fully available without restriction?	
**Describe where the data may be found in full sentences. If you are copying our sample text, replace any instances of XXX with the appropriate details.**<br><br>• If the data are **held or will be held in a public repository**, include URLs, accession numbers or DOIs. If this information will only be available after acceptance, indicate this by ticking the box below. For example: *All XXX files are available from the XXX database (accession number(s) XXX, XXX.).*<br>• If the data are all contained **within the manuscript and/or Supporting Information files**, enter the following: *All relevant data are within the manuscript and its Supporting Information files.*<br>• If neither of these applies but you are able to provide **details of access elsewhere**, with or without limitations, please do so. For example:<br><br>*Data cannot be shared publicly because of [XXX]. Data are available from the XXX Institutional Data Access / Ethics Committee (contact via XXX) for researchers who meet the criteria for access to confidential data.*<br><br>*The data underlying the results presented in the study are available from (include the name of the third party*	All relevant data are within the manuscript and its Supporting Information files.

and contact information or URL).<br>• This text is appropriate if the data are owned by a third party and authors do not have permission to share the data.<br><br>* typeset	
Additional data availability information:	

Dear Editor,

Thank you for considering our work and give us an opportunity. We modified the content following you and the reviewers' comments, and outline every change point by point. Looking forward to hearing from you as soon as possible.

Yours sincerely,

Lihua Zhao

Correspondence: zhongkai99@sina.com (Z. Zhang.)

Manucript without track changes Click here to access/download;Manuscript;Manucript - 2021.11.14.docx

Functional analysis of the nonstructural protein NSs of tomato zonate spot virus

Jing-Li[1¶], Si-Chen[1, 2¶], Run-Shuang Qiu[2], Li-Zhen Zhang[2], Yue Chen[2], Xue Zheng[2], Ting-Ting Li[2], Li-Hua Zhao[2*], Zhong-Kai Zhang[2*]

1 Life Science College, Southwest Forestry University, Kunming, China

2 Institute of Biotechnology and Germplasm Resources, Yunnan Academy of Agricultural Sciences Yunnan Provincial Key Lab of Agricultural Biotechnology, Key Lab of Southwestern Crop Gene Resources and Germplasm Innovation, Ministry of Agriculture, Kunming, China

¶ These authors contributed equally to this work.

* Correspondence: 356429686@qq.com; zhongkai99@sina.com

Abstract

Tomato zonate spot virus (TZSV), a member of the genus *orthotospovirsus*, causes severe damage to vegetables and ornamental crops in southwest China. The NSs protein is an RNA silencing suppressor in various *orthotospovirsus* like TZSV, but its mechanism and role in virus infection are poorly understood. Here, we observed that an NSs-GFP fusion protein was transiently expressed on the plasma membrane and Golgi bodies in *Nicotiana benthamiana* plants. The TZSV *NSs* gene was silenced and infiltrated into *N. benthamiana* and *N. tabacum* cv. K326. RT-qPCR and Indirect enzyme-linked immunosorbent assay (ID-ELISA) showed that the transcription and the protein expression of the *NSs* gene were inhibited by more than 90.00%, and the symptoms on silenced plants were alleviated. We also found that the expression of the *Zingipain-2-like* gene significantly decreased when the *NSs* gene was silenced, resulting in co-localization of the NSs-GFP and the Zingipain-2-like-mCherry fusion protein. The findings of this study provide new insights into the mechanism of silencing suppression by NSs, as well as its effect on systemic virus infection, and also support the theory of disease resistance breeding and control and prevention of TZSV in the field.

Introduction

Tomato zonate spot virus (TZSV), the dominant species, which belongs to the *orthotospovirus* genus of the *Bunyaviridae* family in Yunnan Province, is transmitted by thrips [1]. In recent years, TZSV has been prevalent in Yunnan, Guizhou, Guangxi, and other regions of China and Southeast Asia. TZSV infection is often associated with severe disease symptoms, including concentric rings and spots on fruits and necrosis of leaves. TZSV has a very broad host range, infecting more than 20 types of economically important crops and weed species, which belong to 7 families [2, 3]. This prevalence has not only led to production losses and quality problems for important vegetable and ornamental plants in Southwest China but also seriously threatened economically important crops for local farmers [4].

TZSV consists of spherical, enveloped particles that become distributed in the cytoplasm and the endoplasmic reticulum in mesophyll cells [1, 5]. Like other members of the genus *orthotospovirus*, TZSV contains a tripartite genome consisting of large (L), medium (M), and small (S) segments. The L RNA segment is negative and encodes the RNA-dependent RNA polymerase (RdRp). The M RNA segment encodes the nonstructural NSm protein and the viral glycoprotein precursor (Gn/Gc), while S RNA encodes the nonstructural NSs protein and the nucleocapsid protein N, and the N protein is responsible for the envelope formation of the viral genome RNA [6, 7].

The NSs protein is an RNA-silencing suppressor encoded by orthotospoviruses, such as TZSV, tomato spotted wilt orthotospovirus (TSWV), groundnut ringspot virus (GRSV), and tomato yellow ring virus (TYRV) [1, 8–9]. The silencing suppressor of TSWV is the multifunctional NSs protein that is necessary for systemic movement in plants and can influence the emission of plant volatiles and suppress the JA-regulated plant defenses, resulting in the enhanced attractiveness of plants to flower thrips (*Frankliniella occidentalis*). NSs can also functionally replace potyviral HC-Pro and promote systemic infection and symptom development by suppressing antiviral RNA silencing [6, 10–12]. The NSs protein of watermelon silver mottle virus (WSMoV) has the function of transmission of the virus by *T. palmi* [13]. The NSs protein of TZSV activates a hypersensitive response in resistant plants and could interact with the VDAC protein in F. *ocicdentalis*, regulating the transmission of the persistent-propagative plant

viruses [14]. The mechanisms of RNA-silencing suppression in TZSV by NSs and its role in virus infection need further exploration.

Virus-induced gene silencing (VIGS) is a method to study the functions of plant and pathogen genes by the agroinfiltration or biolistic inoculation of plants. VIGS has been successfully used to investigate gene function and disease resistance. Silencing of the *LeCTR1* gene in tomatoes led to an accumulation of ROS and increased the expression of *NPR1*, *PR1*, *PR5*, and *AOS2* genes to prevent the *tomato leaf curl virus* (ToLCV) infection [15]. When the *H2B* and *CoiI* genes in *N. benthamiana* were silenced, the contents of the phytohormone salicylic acid (SA) and jasmonic acid (JA) increased, and the infection with potato virus X (PVX) and (TSWV) was inhibited [16, 17]. Macharia et al. found that silencing of the *NbHYPK* and *ATG8* genes could enhance autophagocytosis and help combat the TMV infection [18]. The *N* gene of TSWV was inserted into the TRV vector to further study the gene function, but there have been no studies on inserting TZSV genes into VIGS vectors [19]. In this study, *NSs* gene was silenced by constructing the TRV-pTV00 vector and then infiltrated into *N. benthamiana* and *N. tabacum* cv. K326. RT-qPCR and ID-ELISA assays showed that *NSs* gene transcription and protein expression were inhibited more than 90.00%, and the symptoms in the silenced plants were alleviated. We also observed that the expression of the *Zingipain-2-like* gene significantly decreased when *NSs* gene was silenced. Furthermore, the results showed the co-localization expression of NSs-GFP and Zingipain-2-like-mCherry fusion protein on the plasma membrane in *Nicotiana benthamiana* plants. This is the first report using the TRV VIGS system to analyze the functions of the TZSV gene, which have important new implications for mechanistic studies of the suppression of gene silencing by NSs and their effects on systemic infection by the virus.

Materials and Methods

Materials

The pTV00, pBINTRA, and pTV00-PDS vectors were provided by Professor Jianqiang Wu's laboratory at the Kunming Institute of Botany, Chinese Academy of

Sciences (KIB CAS) [20]. The vector of pCAMBIA-GFP, pBI121-mCherry were obtained from our lab in the institute of biotechnology and germplasm resources. The TZSV YN-Chili isolate was collected from the infected tomato field in Yuanmou, Yunnan Province, China, and maintained on *N. benthamiana* [1]. *N. tabacum* cv. K326 and *N. benthamiana* were cultivated at the Yunnan Academy of Agricultural Science. Primers were designed using the Primer 5 Design Program based on the sequences of TZSV *NSs* gene and *Zingipain-2-like* gene published in the NCBI database (registration number: EF552433.1, LOC107763929) (Table S1).

TZSV inoculation

The frictional inoculation method was used to artificially infect *N. tabacum* cv. K326 and *N. benthamiana* plants at the six-leaf stage. TZSV-infected *N. benthamiana* leaves were homogenized in the PBS buffer (100 mg/mL) containing 137 mM NaCl, 1 mM KH_2PO_4, 8 mM$Na_2HPO_4 \cdot 12H_2O$, and 3 mM KCl, and thereafter the homogenate was applied uniformly to 3 leaves per plant (1 mL). Ten minutes after the TZSV inoculation, the inoculated plant leaves were rinsed with ddH_2O. Plant leaves inoculated with PBS buffer were used as controls, and five replicates were used for each sample. Five days after inoculation, symptoms appeared.

RNA extraction and RT-PCR

According to the instructions provided in the RNA extraction kit (Roche, America), the total RNA of plant leaves was extracted. The first-strand cDNA synthesis kit was used for reverse transcription (TransGen, China). Q5 High-Fidelity DNA Polymerase (NEB, England) was used to amplify the target fragments. The PCR solution consisted of 1 µL of cDNA, 10 µL of 10× EasyTaq Buffer, 1 µL of 2.5 mM dNTPs, 2.5 µL of forward and reverse primers, and 0.5 µL of Q5 DNA polymerase; ddH_2O was added to the solution to obtain the final volume of 50 µL. The PCR conditions were as follows: 35 cycles of denaturation at 98 °C for 40 s, 98 °C for 10 s, 60 °C for 20 s, and 72 °C for 30 s, followed by 72 °C for 2 min. The amplified products were subsequently analyzed using a UVP gel-imaging system.

Plasmid constructs

(i) PCR was used to amplify the desired fragments with specific primers (Table S1) using cDNA prepared from the plant tissues inoculated with TZSV. The target PCR fragments were excised, and DNA was extracted using an appropriate kit (AxyGen, America). The amplified fragments of the *NSs* gene and pTV00 vectors were digested by the restriction enzymes *BamHI* and *HindIII* according to the manufacturers' instructions, and the purified products of the *NSs* sequence were inserted into the pTRV-pTV00 vectors using T4 DNA ligase (NEB, England). The fragments of the to-be-silenced *NSs* gene were cloned in the pTV00 vectors. The vectors were then transformed into competent cells of *E. coli* strain DH5α. The selected positive clones were transferred to Guangzhou Huada Gene Technology Co., Ltd., for sequencing. Plasmids with the correct sequence were used to transform *A. tumefaciens* electrocompetent cells.

(ii) The sequences of *NSs* and *Zingipain-2-like* genes were amplified from the total RNA isolated from tobacco plants infected with TZSV using reverse transcription-PCR (RT-PCR) and the special primers (Table S1). The PCR fragments of *NSs* and *Zingipain-2-like* genes were digested with endonuclease and inserted into vector of pCAMBIA-GFP and pBI121-mCherry to obtain pCAMBIA-NSs -GFP and pBI121-Zingipain-2-like-mCherry, respectively. The Golgi marker ST52-mCherry was amplified from the total RNA isolated from both tobacco and Arabidopsis [21].

Infiltration of the VIGs vector in Tobacco Leaves

The pTRV-pTV00-NSs constrcture was transformed into *Agrobacterium tumefaciens* strain GV3101 by electroporation. For every 1 mL of the inoculation solution, 2.5 mL of the liquid YEP culture plus 2.5 mL of pBINTRA liquid YEP culture were used. For each construct, the final inoculation solution was made by mixing equal volumes of the resuspended *Agrobacterium* carrying the pTRV-pTV00-NSs construct and pBINTRA.

Inoculation was performed using a 1-mL syringe, and the inoculation solution was pressure injected into 3 individual leaves per plant. Inoculation of pTRV-pTV00-NSs construct was performed using a 1-mL syringe, and the inoculation solution of 1 mL was pressure injected into individual leave and per-plant was injected 3 leaves. Phytoene

dehydrogenase gene (*PDS*) as an indicator gene, the plant leaves will turn white when it was silenced, so the leaves injected with pTRV-pTV00-PDS construct becoming bleached at approximately 10–14 days, the leaves performed with pTRV-pTV00-NSs construct inoculated with TZSV. Plants injected with pTRV-pTV00 and inoculated with TZSV alone served as positive controls. At approximately 5–10 days post-inoculation, samples were collected and healthy plants were used as blank controls.

Plants injected with pTRV-pTV00 and inoculated with TZSV alone served as positive controls. At approximately 5–10 days post-inoculation, samples were collected and healthy plants were used as blank controls.

Confocal laser scanning microscopy and co-localization

The leaf epidermis was dissected from the areas of the agroinfiltrated *N. benthamiana* leaves and placed in water between two coverslips. The confocal images were captured with the inverted TCS SP8 and 10× water immersion objective lenses. GFP was excited at a wavelength of 488 nm, and emissions were captured at 497–520 nm. Moreover, mCherry was excited at a wavelength of 561 nm and emissions were captured at 585–615 nm. Images were processed using the TCS SP8 and Adobe (San Jose, CA, USA) Photoshop.

RT-qPCR

The sequences of the *NSs* gene were inserted into the pMD18-T vector, and the plasmids were diluted 10-fold with a gradient to obtain plasmids with 10-fold dilution series (10^{-1},10^{-2},10^{-3},10^{-4},10^{-5},10^{-6}), and then used as calibrators to construct a standard curve. The reaction system consisted of the components including 1 μL of cDNA, 2.5 μL of forward and reverse primers,1 0 μL of FastStart Universal SYBR Green Master (Rox), and 4 μL of ddH_2O. The reaction conditions were as follows: initial denaturation step at 95 °C for 10 min, followed by 40 cycles of 95 °C for 10 s, 57 °C for 30 s, and 72 °C for 30 s.

ID-ELISA

The contents of the proteins were tested by ID-ELISA according to the instructions

for antibodies to determine the antiviral activity of VIGS. Leaves (0.2 g) were homogenized using a mortar and pestle and diluted 1: 3 in a PBS buffer. Crude extracts (100 μL) were added into ELISA plate wells and incubated at 37 °C for 2 h. The plate was then washed with PBST buffer. TZSV NSs rabbit antibodies were diluted in a conjugation buffer, and afterward, 100 μL of goat anti-rabbit IgG-AP conjugate (Sigma, USA) was added to each well. The color-developing solution was dissolved in p-nitrophenyl phosphate disodium hexahydrate (Sigma-Aldrich) in substrate buffer to obtain a final concentration of 1 mg/mL. The absorbance was determined at 405 nm using the ELx 808 microplate ELISA reader (Bio-Tek, USA). Healthy leaves were used as negative controls, and TZSV-infected leaves were used as positive controls. The PBS buffer was used as a blank control.

Results

NSs was localized to the PM and Golgi bodies

Previous studies have shown that confocal laser scanning microscopy (CLSM) was used to analyze the protein localized in living cells [22]. *N. benthamiana* is also a model plant species to assess the subcellular localization of viral proteins. To characterize the subcellular targeting of the NSs in plant cells, the recombinant NSs-GFP (green fluorescent protein) was first transiently expressed in leaf epidermal cells of *N. benthamiana* by agroinfiltration. NSs-GFP was then detected to be associated with the plasma membrane structures by CLSM (Fig 1 A-C). To determine whether the NSs-GFP bodies are colocalized with the Golgi stacks, we also checked the localization of NSs-GFP for Golgi bodies using the marker ST52-mCherry [23]. At 48 h after agroinfiltration on *N. benthamiana*, we found that the NSs-GFP bodies are colocalized with the Golgi stacks (Fig 1 D-F), suggesting that the NSs-GFP protein was targeted to the Golgi apparatus.

Silencing of the *NSs* gene

TRV vector was widely used to study the interactions between viruses and hosts and the functions of plant genes [24]. In the present study, specific primers

(TZVNSsF1/TZVNSsR1) containing *BamHI* and *HindIII* restriction enzyme recognition sites were used to amplify the *NSs* gene fragments (Fig S1), and the DNA was inserted into the pEASY-Blunt-Zero vector (TransGen, Beijing) for sequencing to ensure that the base sequences were not mutated.

The copies of the *NS*s gene were analyzed by RT-qPCR. The fragments of the *NSs* gene were amplified and inserted into the pEASY-T1 Simple vector (Trans, Beijing). The concentration and the $OD_{260/280}$ value for the recombinant plasmid containing the *NSs* gene were 225.82 ng/µL and 1.83, respectively. The plasmid DNA with gradient dilutions of 10^{-1} to 10^{-6} was used as a template. Standard curves and amplification curves of RT-qPCR data for the *NSs* gene were obtained by automatic analysis performed by the software system. The standard curve equation of the *NSs* gene was Y =-3.444X+34.42, and the amplification efficiency and the correlation coefficient were 90.90% and 0.998, respectively. The results showed that the plasmid DNA could be used as a calibration product to determine the copy numbers of the gene.

The *PDS* gene was used as a positive control to ensure the success of silencing. The leaves of *N. benthamiana* without this gene were bleached. However, there were no phenotypic changes in leaves of the *N. tabacum* cv. K326, despite the fact that these species belong to the same genus. TZSV was inoculated on the two species tobaccos that the plants were injected with pTRV-pTV00-NSs construct and positive control plants (inoculated only with TZSV), respectively, when the leaves in the veins of *N. benthamiana* turned white injecting with pTRV-pTV00-PDS construct. After 5 days of inoculation with TZSV, the shrinkage also appeared on the leaves of *N. benthamiana* plants injected with the pTRV-pTV00-NSs vector before inoculation with TZSV (Fig 2A). Severe leaf shrinkage also occurred in positive control plants (inoculated only with TZSV) (Fig 2B); however, for *N. tabacum* cv. K326, the symptoms in the VIGS plants and positive control plants were not different. To further determine the effects of the *NSs* gene, its transcription level was measured by RT-qPCR and found to be significantly decreased compared to that of the positive control (inoculated only with TZSV) in both hosts. The silencing efficiencies of the TZSV *NSs* gene were 99.16% in *N. benthamiana* and 92.24% in *N. tabacum* cv. K326 (Fig 3A, B). The results indicate

that the pTRV-pTV00-NSs VIGS vector was successfully constructed, and the *NSs* gene might be associated with TZSV infection.

Inhibition of the NSs protein expression

The NSs protein levels were measured by ID-ELISA at 3, 5, 7, and 9 days post-inoculation with TZSV, and found to be significantly decreased in leaves of *N. benthamiana* and *N. tabacum* cv. K326 plants treated with the pTRV-pTV00-NSs construct and inoculated with TZSV compared to those in positive plants at 7 and 9 days (Table 1). The results showed that the expression of the *NSs* protein in both *N. benthamiana* and *N. tabacum* cv. K326 hosts was inhibited.

The dependence of *NSs* gene silencing on the *zingipain-2-like* gene

The *Zingipain-2-like* gene was a homocysteine protease, which possesses cysteine-type endopeptidase activity and participates in the regulation of plant-type hypersensitive response [25, 26]. To confirm whether the host factor for the *Zingipain-2-like* gene made closely relationship with RNA silencing suppressing by the NSs gene of TZSV, the expression of the *Zingipain-2-like* gene performed by *NSs*, the silencing suppressor in plants, was detected by RT-qPCR assay, and found to be significantly decreased in the *NSs* gene-silenced plants compared to positive plants, compared with the CK, expression of the Zingipain-2-like gene was up-regulated in both positive and NSs-silenced plants infected by TZSV plants, but higher in the positive plant. The rationale behind these data could be that *Zingipain-2-like* gene expression was induced by NSs (Fig 3 C).

To further investigate whether NSs and the Zingipain-2-like protein were colocalized and to identify the specific structures in living cells, we transiently expressed the recombinant NSs-GFP (green fluorescent protein) and Zingipain-2-like-RFP (red fluorescent protein) in leaf epidermal cells of *N. benthamiana* by agroinfiltration. The co-expression of NSs-GFP with the Zingipain-2-like-RFP confirmed the co-localization of NSs and Zingipain-2-like at the plasma membrane by the confocal laser scanning microscope (CLSM) (Fig 4 A-C). Our results clarified that the RNA silencing suppressor (NSs) had a close relationship with Zingipain-2-like

when TZSV infected the plants.

Discussion

The importance of NSs for tospoviral infection in plants was first discovered in the early 1990s. A higher virulence of TSWV isolates and more severe symptoms were observed with the elevated levels of NSs expression [27]. The TSWV NSs protein acts as a suppressor of RNA silencing through binding small and long dsRNA and suppresses short and long-distance viral accumulation and movement [28]. It also represents the Avr factor of the *Tsw* resistance gene in pepper [29, 30]. NSs of TSWV directly interact with *MYC2*, a regulator of the JA signaling, to disable JA-mediated activation of terpene synthase genes and attenuate host defenses, increasing the attractiveness of the plants to thrips, and thus transmitting the disease [31]. So far, the ability of NSs of TSWV to counteract defense mechanisms mediated by RNA silencing in plants has been demonstrated; however, the mechanism of RNA silencing suppression by NSs in TZSV and its role in virus infection are not yet clear. In the present study, the accumulation of the virus decreased and symptoms were alleviated when the *NSs* gene was silenced. The expression of the *Zingipain-2-like* gene was found to be significantly decreased in the *NSs* gene-silenced plants compared to positive plants, compared with the CK, expression of the Zingipain-2-like gene was up-regulated in both positive and NSs-silenced plants infected by TZSV plants, but higher in the positive plant. We first revealed that the *Zingipain-2-like* gene might be associated with this function.

The innate immune system of plants has two different layers, including microbe-associated (MAMP) or pathogen-associated molecular pattern (PAMP)-triggered immunity (PTI) and effector-triggered immunity (ETI). PTI is mediated by the corresponding membrane-anchored pattern recognition receptors (PRRs) in plants, which serve as the first line of defense against the pathogen. Many plant viruses and their encoded proteins that could inhibit PTI, like the NSs protein of TSWV that suppressed the production of reactive oxygen species (ROS), have been reported [30, 32, 33]. In this study, we found that NSs and Zingipain-2-like were colocalized at the

plasma membrane; the expression of the *Zingipain-2-like* gene significantly decreased in *NSs* gene-silenced plants compared to positive plants, and Zingipain-2-like took part in the regulation of hypersensitive response, suggesting that NSs and Zingipain-2-like might be associated with the activation of PTI-like responses.

In this study, *N. benthamiana* was used as a model plant, and the ability of the NSs protein of TZSV to target Golgi bodies in plant cells was observed for the first time. The targeting of the virus glycoproteins to the Golgi apparatus plays a pivotal role in the formation of enveloped spherical particles of the viruses belonging to the Bunyaviridae family [34–36]. However, the reason for the facilitation of the formation of enveloped spherical particles by the NSs protein remains to be extensively investigated.

In this study, TRV vectors were used to construct VIGS vectors of the *NSs* gene for the analysis of their functions. The results of RT-qPCR, as well as the plant disease symptoms, showed that the gene replication was inhibited up to 90%. ID-ELISA showed that the protein contents also significantly decreased. The high efficiency of gene silencing can be verified by sampling and testing immediately at the onset of the disease. At the same time, temperature also had effects on the silencing phenotypes in plants [37]. In this experiment, the temperature was strictly controlled, and thus, the gene was silenced at relatively high levels, and the duration was relatively long. In this study, the *N. benthamiana* leaves were bleached, but there were no phenotypic changes in leaves of *N. tabacum* cv. K326, despite the fact that both species belong to the same genus, indicating that the TRV-VIGS vector exhibited differing sensitivities to different host species. TRV has a wide range of hosts, with a significant difference in sensitivity to TRV between species and cultivars [38, 39]. For instance, TRV sensitivity testing was carried out on 21 gerbera cultivars, and the results revealed that only 5 cultivars showed photobleached PDS-silencing symptoms on newly developed leaves [40]. The VIGS method can be used for reverse genetics studies and the analysis of the functions of unknown genes.

In summary, our results revealed that NSs, a suppressor of RNA silencing in TZSV, was localized to the PM and Golgi bodies and might also be associated with

Zingipain-2-like to activate PTI-like responses using VIGS and subcellular localization prediction.

Acknowledgements

The authors would like to thank Professor Jianqiang Wu's laboratory KIB. CAS. for providing the VIGS vectors. We also thank the Yunnan Academy of Agricultural Sciences Yunnan Provincial Key Lab of Agricultural Biotechnology for equipment support of the confocal microscope.

Supporting information

S1Table. Special primers. The primers were used to construct the VIGS, fluorescence labeling, and RT-qPCR vectors.

S1 File. Amplication of the *NSs* gene sequence for VIGs.

Author Contributions

Conceptualization: Li-Hua Zhao.

Formal analysis: Zhong-Kai Zhang.

Methodology: Ting-Ting Li, Si-Chen, Yue Chen, Run-Shuang Qiu

Resources: Li-Hua Zhao, Zhong-Kai Zhang.

Supervision: Li-Hua Zhao

Validation: ZhongKai-Zhang.

Writing – original draft: Jing-Li, Si-Chen, Li-Hua Zhao.

Writing – review & editing: Jing-Li, Li-Hua Zhao, Li-Zhen Zhang, Xue Zheng, Zhong-Kai Zhang.

Funding

This study was supported by National Natural Science Foundation of China (No. U1802235, 31960531), Yunnan Fundamental Research Projects (202101AT070269).

Reference

1. Dong JH, ChengXF, Yin YY,Fang Q, Ding M, Li TT, Zhang LZ, Su XX, McBeath JH, Zhang ZK. Characterization of tomato zonate spot virus, a new *tospovirus* in China. Arch Virol. 2008; 153(5): 855-864.

2. Liu Y, Huang CJ, Tao XR, Yu HQ. First report of tomato zonate spot virus in *Iris tectorum* in

China. Plant dis. 2015; 99(1): 164-164.

3. Zhu M, Jiang L, Bai BH, Zhao WY, Chen XJ, Li J, Yong Liu 2, Chen ZQ, Wang BT, Wang CL, Wu Q, Shen QH, Dinesh-Kumar SP, Tao XR. The intracellular immune receptor Sw-5b confers broad-spectrum resistance to Tospoviruses through recognition of a conserved 21-amino-acid viral effector epitope. Plant Cell. 2017; 29(9): 2214-2232.4. Cai JH, Qin BX, Wei X P, Huang J, Zhou WL, Lin BS, Yao M, Hu ZZ, Feng ZK, Tao XR. Molecular identification and characterization of tomato zonate spot virus in tobacco in Guangxi, China. Plant dis. 2011; 95(11): 1483-1483.

5. Niu YB, Wang DF, Cui LY, Wang BX, Pang XJ, Yu PX. Monoclonal antibody-based colloid gold immunochromatographic strip for the rapid detection of tomato zonate spot tospovirus. Virol J. 2018; 15(1): 15.

6. Takeda A, Sugiyama K, Nagano H, Mori M, Kaido M, Mise K, Tsuda S, Okuno T. Identification of a novel RNA silencing suppressor, NSs protein of tomato spotted wilt virus. FEBS Lett. 2002; 532(1-2): 75-79.

7. Zhang ZK, Zheng KY, Dong JH,Fang Q, Hong J, Wang XF. Clustering and cellular distribution characteristics of virus particles of tomato spotted wilt virus and tomato zonate spot virus in different plant hosts. Virol. J. 2016; 13(1): 11.

8. Margaria P, Miozzi L, Rosa C, Axtell MJ, Pappu HR, Turina M. Small RNA profiles of wild-type and silencing suppressor-deficient tomato spotted wilt virus infected *Nicotiana benthamiana*. Virus Res. 2015; 208: 30-8.

9. Schnettler E, Hemmes H, Huismann R, Goldbach R, Prins M, Kormelink R. Diverging affinity of tospovirus RNA silencing suppressor proteins, NSs, for various RNA duplex molecules. J Virol. 2010; 84(21): 11542-54.

10. Margaria P, Bosco L, Vallino M, Ciuffo M, Mautino GC, Tavella L, Turina M. The NSs protein of tomato spotted wilt virus is required for persistent infection and transmission by *Frankliniella occidentalis*. J. Virol. 2014; 88: 5788-5802.

11. Garcia-Ruiz H, Sergio M, Peralta G, Patricia A. Maxwell H. Tomato spotted wilt virus NSs protein supports infection and systemic movement of a potyvirus and is a symptom determinant. Viruses. 2018; 10: 129.

12. Du J, Song XY, Shi XB, Tang X, Chen JB, Zhang ZH, Chen G, Zhang Z, Zhou XG, Liu Y,

Zhang DY. NSs, the silencing suppressor of tomato spotted wilt orthotospovirus, interferes with JA-regulated host terpenoids expression to attract *Frankliniella occidentalis*. Frontiers in Microbiology. 2020; 11: 590451.

13. Mou DF, Chen WT, Li WH, Chen TC, Tseng CH, Huang LH, Peng JC, Yeh SD, Tsai CW. Transmission mode of watermelon silver mottle virus by Thrips palmi. PloS ONE. 2021; 16(3): e0247500.
14. Zhao XY, Chen JB, Wang SG, Zhang XL, Mu Y, Wei H, Zhao LH, Chen YD, Zheng X, Chen Y, Zheng LM. Zhang J. Screening and identification of the protein interacting with the NSs protein of tomato zonate spot virus in *Franiklinella ocicdentalis* (Thysanoptera: Thripidae) by yeast two-hybrid technique. Acta Entomologica Sinica. 2020; 63(6): 735-743. (in Chinese)
15. Chandan RK, Singh AK, Patel S, Swain DM, Tuteja N, Jha G. Silencing of tomato *CTR1* provides enhanced tolerance against tomato leaf curl virus infection. Plant Signal Behav. 2019; 14(3): e1565595.
16. Yang X, Lu YW, Zhao X, Jiang LL, Xu SC, Peng JJ, Zheng HY, Lin L, Wu YH, MacFarlane S, Chen JP, Yan F. Downregulation of nuclear protein H2B induces salicylic acid mediated defense against PVX infection in *Nicotiana benthamiana*. Front Microbiol. 2019; 10: 1000.
17. Zhao LH, Hu ZH, Li SL, Zhou XP, Li J, Su XX, Zhang LZ, Zhang ZK, Dong JH. Diterpenoid compounds from *Wedelia trilobata* induce resistance to tomato spotted wilt virus via the JA signal pathway in tobacco plants. Sci Rep. 2019; 9: 2763.
18. Macharia MW, Wilfred YZ, Das PP, Naqvi NI, Wong SM. Proximity-dependent biotinylation screening identifies *NbHYPK* as a novel interacting partner of *ATG8* in plants. BMC Plant Biol. 2019; 19: 326.
19. Qiu RS, Zhao LH, Zhang XM, Zhang J,Zheng X, Li J, Zhang ZK. Construction of a VIGS vector containing *N* gene derived from tomato spotted wilt orthospovirus. Acta phytopathologicasinica. 2020; 50(6): 766-733.
20. Ratcliff F, Martin-Hernandez AM, Baulcombe DC. Tobacco rattle virus as a vector for analysis of gene function by silencing. Plant J. 2001; 25(2): 237-245.
21. Dupas SJC, Nebenfu A, Boulaflous A, Gueye MLF, Plasson C, Hawes C, Driouich A, Faye L, Gomord V. Plant N-glycan processing enzymes employ different targeting mechanisms for their spatial arrangement along the secretory pathway. The Plant Cell. 2006; 18: 3182-3200.

22. Feng ZK, Xue F, Xu M, Chen XJ, Zhao WY, Garcia-Murria MJ, Mingarro I, Liu Y, Huang Y, Jiang L, Zhu M, Tao XR. The ER-membrane transport system is critical for intercellular trafficking of the NSm movement protein and tomato spotted wilt tospovirus. PLoS Pathog. 2016; 12(2): e1005443.

23. Nelson BK, Cai X, Nebenfuhr A. A multicolored set of in vivo organelle markers for colocalization studies in *Arabidopsis* and other plants. Plant J. 2007; 51: 1126-1136.

24. Tavares-Esashika ML, Campos RNS, Blawid R, da Luz LL, Inoue-Nagata AK, Nagata T. Characterization of an infectious clone of pepper ringspot virus and its use as a viral vector. Arch Virol. 2020; 165(2): 367-375.

25. Khanna-Chopra R, Srivalli B, Ahlawat YS. Drought induces many forms of cysteine proteases not observed during natural senescence. Biochemical & Biophysical Research Communications. 1999; 255(2): 324.

26. Rungsaeng P, Sangvanich P, Karnchanatat A. Zingipain, a ginger protease with acetylcholinesterase inhibitory activity. Appl Biochem Biotechnol. 2013; 170: 934-950.

27. Kormelink R, Kitajima EW, De Haan P, Zuidema D, Peters D, Goldbach R. The nonstructural protein (NSs) encoded by the ambisense S RNA segment of tomato spotted wilt virus is associated with fibrous structures in infected plant cells. Virology. 1991; 181: 459–468.

28. Hedil M, Kormelink R. Viral RNA silencing suppression: the enigma of Bunyavirus NSs proteins. Viruses. 2016; 8: 208.

29. De Ronde D, Pasquier A, Ying S, Butterbach P, Lohuis D, Kormelink R. Analysis of tomato spotted wilt virus NSs protein indicates the importance of the N-terminal domain for avirulence and RNA silencing suppression. Mol. Plant Pathol. 2014; 15: 185-195.

30. Huang C, Liu Y, Yu HQ, Yuan C, Zeng JM, Zhao L, Tong ZJ, Tao XR. Non-structural protein NSm of tomato spotted wilt virus is an avirulence factor recognized by resistance genes of tobacco and tomato via different elicitor active sites. Viruses. 2018; 10: 660.

31. Wu XJ, Xu SH, Zhao PZ, Zhang X, Yao XM, Sun YM, Fang RX, Ye Jian. The *Orthotospovirus* nonstructural protein NSs suppresses plant MYC-regulated jasmonate signaling leading to enhanced vector attraction and performance. PLoS Pathog 2019; 15(6): e1007897.

32. Hogenhout SA, Van der Hoorn RAL, Terauchi R, Kamoun S. Emerging concepts in effector biology of plant-associated organisms. Mol. Plant Microbe Interact. 2009; 22: 115-122.

33. Gupta R, Min CW, Kim SW, Yoo JS, Moon AR, Shin AY, Kwon SY, Kim ST. A TMT-based quantitative proteome analysis to elucidate the TSWV induced signaling cascade in susceptible and resistant cultivars of *Solanum lycopersicum*. Plants (Basel) 2020; 9(3): 290.

34. Kikkert M, Van LJ, Storms M, Bodegom P, Kormelink R, Goldbach R. Tomato spotted wilt virus particle morphogenesis in plant cells. J. Virol. 1999; 73: 2288-2297.

35. Shi X, Lappin DF, Elliott RM. Mapping the Golgi targeting and retention signal of *Bunyamwera* virus glycoproteins. J. Virol. 2004; 78: 10793-10802.

36. Yao M, Liu XF, Li S, Xu Y, Zhou YJ, Zhou XP, Tao XR. Rice Stripe Tenuivirus NSvc2 Glycoproteins targeted to the Golgi body by the N-Termina transmembrane domain and adjacent cytosolic 24 amino acids via the COP I- and COP II-dependent secretion pathway. Journal of Virology. 2014; 88(6): 3223-3234.

37. Fu DQ, Zhu BZ, ZhuHL, Zhang HX, Xie YH, Jiang WB, Zhao XD, Luo KB. Enhancement of virus induced gene silencing in tomato by low temperature and low humidity. Mol Cells 2006; 21(1): 153-160.

38. Bennypaul HS, Mutti JS, Rustgi S, Kulvinder SG. Virus induced gene silencing (VIGS) of genes expressed in root, leaf, and meiotic tissues of wheat. Funct Integr Genomics. 2012; 12 (1): 143-156.

39. Senthilkumar M, Mysore KS. Tobacco rattle virus-based virus-induced gene silencing in *Nicotiana benthamiana*. Nat Protoc. 2014; 9 (7): 1549-1562.

40. Deng X, Elomaa P, Nguyen CX, Hytönen T, Valkonen JPT, Teeri TH. Virus induced gene silencing for Asteraceae a reverse genetics approach for functional genomics in *Gerbera hybrida*. Plant Biotech. J. 2012; 10(8): 970-97.

Table 1 Detection of NSs protein content with different treatment in both host using ID-ELISA

	pTRV-pTV00-NSs+TZSV				TZSV				pTV00+ TZSV				CK			
Host species	3 d	5 d	7 d	9 d	3 d	5 d	7 d	9 d	3 d	5 d	7 d	9 d	3 d	5 d	7 d	9 d
N. benthamiana	0.207 ± 0.010a	0.214 ± 0.055a	0.244 ± 0.019a	0.201 ± 0.011a	0.219 ± 0.001ac	0.289 ± 0.002ac	0.446 ± 0.008bc	0.369 ± 0.014bc	0.208 ± 0.010ac	0.287 ± 0.005ac	0.396 ± 0.002bc	0.379 ± 0.017bc	0.169 ± 0.009d	0.163 ± 0.013d	0.168 ± 0.014d	0.162 ± 0.013d
N. tabacum cv. K326	0.181 ± 0.009a	0.218 ± 0.007a	0.257 ± 0.006a	0.225 ± 0.062a	0.218 ± 0.006ac	0.282 ± 0.008ac	0.434 ± 0.043bc	0.391 ± 0.011bc	0.217 ± 0.007ac	0.245 ± 0.002ac	0.374 ± 0.010bc	0.368 ± 0.001bc	0.163 ± 0.006d	0.147 ± 0.007d	0.163 ± 0.004d	0.133 ± 0.008d

pTRV-pTV00-NSs +TZSV: infiltrated pTRV-pTV00-NSs construct and inoculated with TZSV; TZSV: positive control and means only inoculated with TZSV; pTV00-TZSV: means infiltrated pTRV-pTV00 vector and inoculated TZSV; CK: negative control and means the leaves with no treatment. All values are means ± SE. Means in a column followed by different letters are significantly different at P ≤ 0.05.

Fig 1 The NSs protein in TZSV is localized at the PM and Golgi bodies

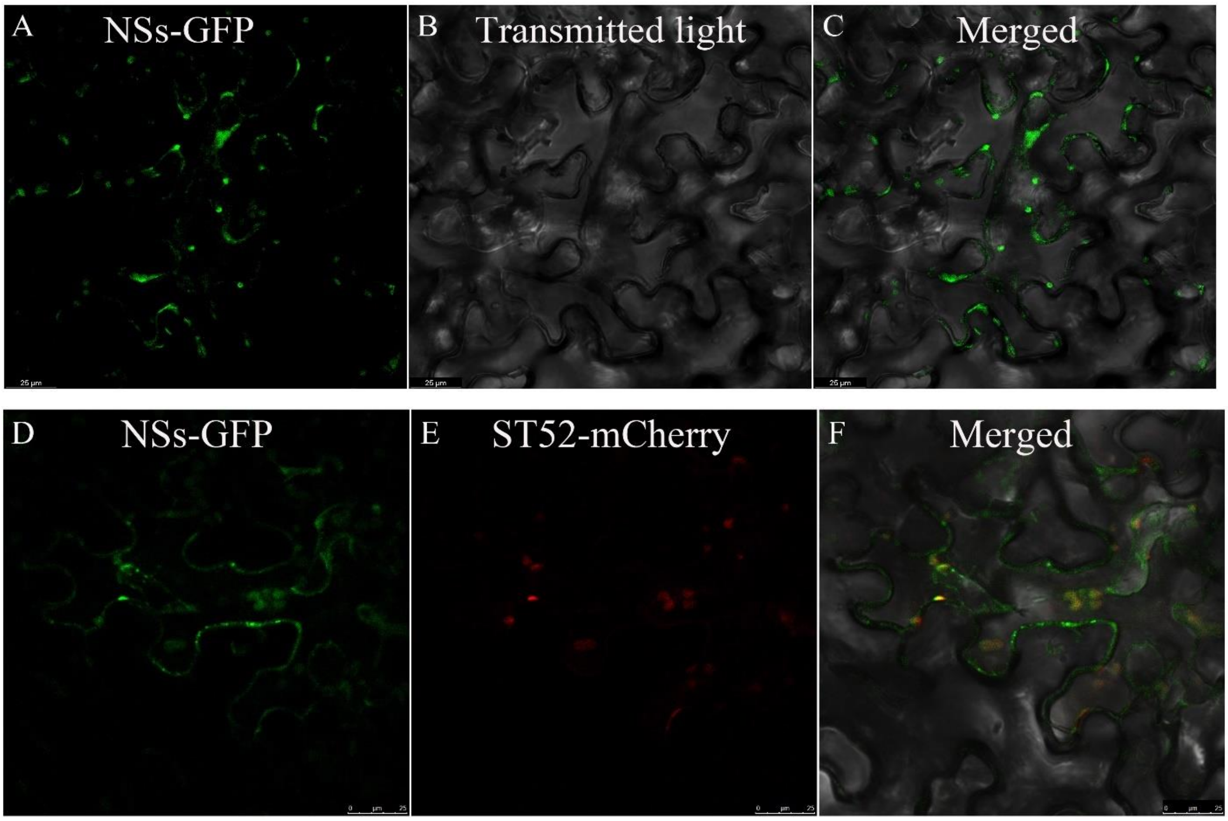

A-C: localization of NSs-GFP at 48 h post infiltration (hpi). Bar, 25 μm. D-F: co-localization of NSs-GFP with Golgi bodies at 48 hpi. Bar, 25 μm. The fluorescence derived from N. *benthamiana*

leaves was monitored using a confocal Leica TCS SP8.

Fig 2 The symptom of TZSV infection on *N. benthamiana*

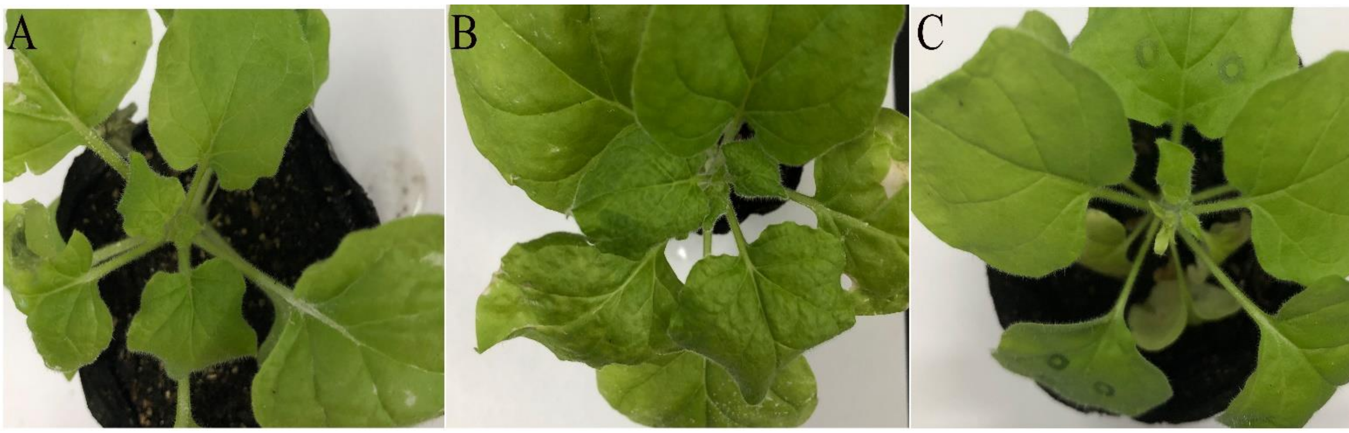

A: infiltrating pTRV-PTV00-NSs construct in the leaves and inoculated with TZSV; B: posivite control (inoculated with TZSV only); C: negative control (the healthy plant)

Fig 3 RT-qPCR detected the transcription of *NSs* and *Zingipain-2-like* genes after *NSs* gene silenced

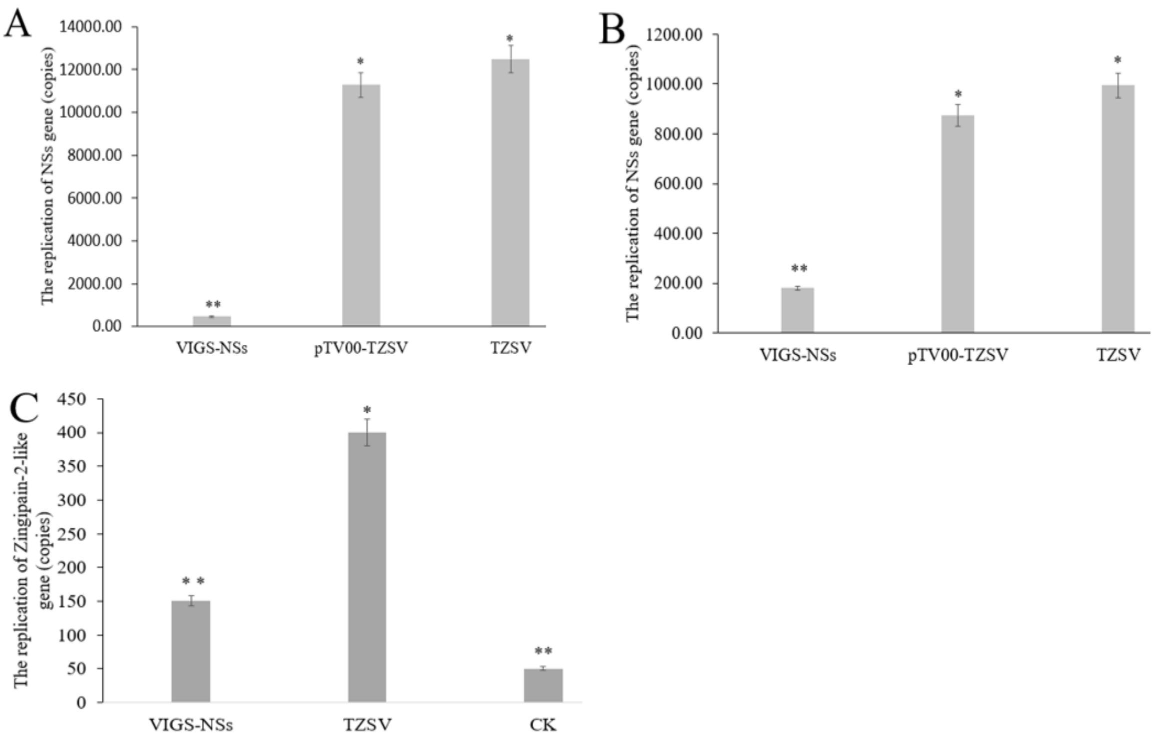

A: the expression of *NSs* gene is detected with different treatment in *Nicotiana Benthamiana* leaves;

B: the expression of *NSs* gene is detected with different treatment in *N. tabacum* cv. K326 leaves;

C: the expression of *Zingipain-2-like* gene is detected in *Nicotiana Benthamiana* leaves; VIGS-NSs: infiltrated pTRV-pTV00-NSs construct and inoculated with TZSV; pTV00-TZSV: infiltrated pTRV-pTV00 vector and inoculated with TZSV; TZSV: positive control and only inoculated with TZSV; CK: negative control (the healthy plant). All values are means ± SE. *means differences are significantly different at $P \leq 0.05$.

Fig 4 The NSs protein in TZSV is co-localized with the Zingipain-2-like protein

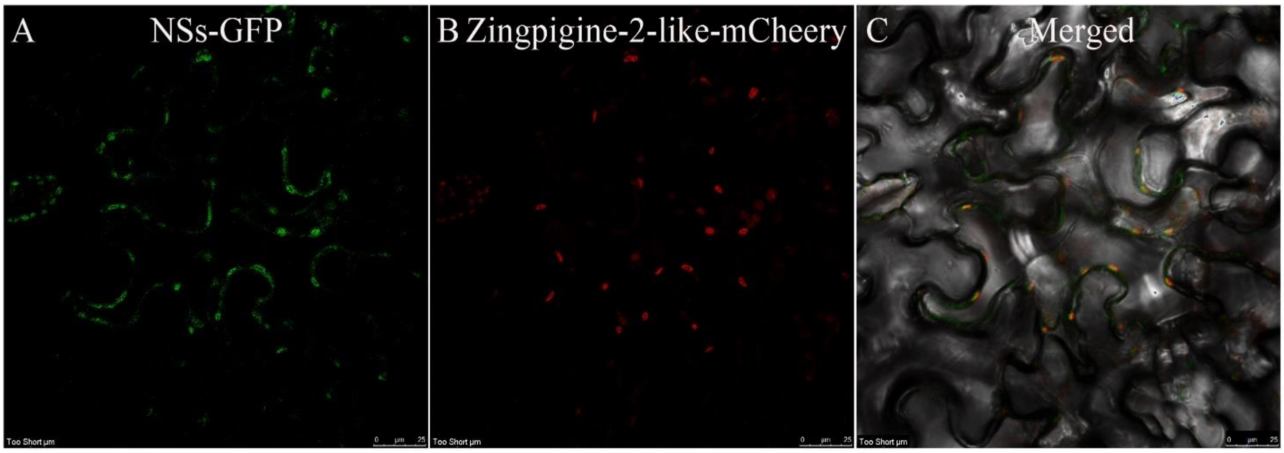

A-C: Colocalization of NSs-GFP with Zingipain-2-like at 48 hpi. Bar, 25 μm. The fluorescence derived from N. *benthamiana* leaves was monitored using a confocal Leica TCS SP8.

Click here to access/download
Supporting Information
Supporting Information -2021.11.14.docx

Click here to access/download

Other

Changes in list-2021.11.14.docx

Functional analysis of~~Analysis function for~~ the~~a~~ nonstructural ~~NSs~~ protein NSs of tomato zonate spot ~~orthotospo~~virus

Jing-Li[1]¶, Si-Chen[1, 2]¶, Run-Shuang Qiu[2], Li-Zhen Zhang[2], Yue Chen[2], Xue Zheng[2], Ting-Ting Li[2], Li-Hua Zhao[2*], Zhong-Kai Zhang[2*]

1 Life Science College, Southwest Forestry University, Kunming, China

2 Institute of Biotechnology and Germplasm Resources, Yunnan Academy of Agricultural Sciences Yunnan Provincial Key Lab of Agricultural Biotechnology, Key Lab of Southwestern Crop Gene Resources and Germplasm Innovation, Ministry of Agriculture, Kunming, China

¶ These authors contributed equally to this work.

* Correspondence: 356429686@qq.com; zhongkai99@sina.com

Abstract

Tomato zonate spot ~~orthotospovirus~~virus (TZSV), a member of the genus *orthotospovirsus*, causes~~causing~~ severe damage to~~reductions of~~ vegetables and ornamental crops in southwest China. The NSs protein is an RNA silencing suppressor in various *orthotospovirsus*~~,~~ like~~such as~~ TZSV, but its~~the~~ mechanism~~ of NSs~~ and~~ its~~ role in virus infection are poorly understood. Here, we ~~showed~~observed that an NSs-GFP fusion protein was transiently expressed on the plasma membrane (PM) and ~~the~~ Golgi bodies in *Nicotiana benthamiana* plants. The~~When~~ TZSV *NSs* gene was silenced and infiltrated into *N. benthamiana* and *N. tabacum* cv. K326. RT-qPCR and Indirect enzyme-linked immunosorbent assay (ID-ELISA)~~ analysis~~ showed that ~~*NSs* gene~~ the ~~replications~~transcription and the protein expression of the *NSs* gene were inhibited by more than 90.00%, and the symptoms on~~of~~ ~~the~~ silenced plants ~~silenced~~ were alleviated. We also found~~showed~~ that the expression of the *Zingipain-2-like* gene significantly ~~significant~~ decreased when the *NSs* ~~genes~~gene was silenced, resulting in co-localization of the NSs-GFP and the Zingipain-2-like-mCherry~~mCheery~~ fusion protein~~ could co-localization expression~~. The ~~results~~findings of this study provide new insights~~insight~~ into the mechanism of silencing suppression by NSs, as well as~~and~~ its effect on ~~virus~~

systemic virus infection, and also support the theory of disease resistance breeding and control~~preventing~~ and prevention of~~controlling~~ TZSV in the field.

Introduction

Tomato zonate spot virus~~orthotospovirus~~ (TZSV), the dominant species, which belongs to the *orthotospovirus* ~~*orthotospovirsus*~~ genus of the *Bunyaviridae* family~~, is the dominant species of *orthospovirus*~~ in Yunnan Province~~, and~~ is transmitted by thrips [1]. In recent years, TZSV has been prevalent in Yunnan, Guizhou, ~~and~~ Guangxi, and ~~in~~ other regions of China and Southeast Asia~~Asian countries~~. TZSV infection is often associated with severe disease symptoms, including concentric rings and~~ring~~ spots on fruits and necrosis of leaves. TZSV has a very broad~~The~~ host range~~of TZSV is very broad~~, ~~and TZSV can infect~~infecting more than 20 ~~kinds~~types of ~~plant species belonging to 7 families, including~~ economically important crops and weed species, which belong to 7 families [2, 3]. This prevalence has not only led to production losses and quality problems for important vegetable and ornamental plants~~plant species~~ in ~~southwest~~Southwest China but also~~and has~~ seriously threatened economically important crops for~~of~~ local farmers [4].

TZSV consists~~consist~~ of spherical, enveloped ~~virus~~ particles ~~and~~that become distributed in the cytoplasm and the endoplasmic reticulum ~~of~~in mesophyll cells [1, 5]. Like other members of the genus *orthotospovir~~s~~us*, TZSV contains a tripartite genome consisting of large (L), medium (M), and small (S) segments. The L RNA segment is negative and encodes the RNA-dependent RNA polymerase (RdRp). The M RNA segment encodes the~~a~~ nonstructural NSm protein and ~~a~~the viral glycoprotein precursor (Gn/Gc), while S RNA encodes ~~a~~the nonstructural NSs protein and ~~a~~the nucleocapsid ~~(N)~~ protein N~~, the NSs protein is a RNA silencing suppressor~~, and the N protein is responsible for the envelope formation of ~~enveloping~~ the viral genome~~genomic~~ RNA [6, 7].

The NSs protein is an RNA-silencing suppressor encoded by ~~viruses in~~ orthotospoviruses, such as TZSV, tomato spotted wilt orthotospovirus (TSWV), groundnut ringspot virus (GRSV), and tomato yellow ring virus (TYRV) [1, 8–9]. The

silencing suppressor of TSWV ~~NSs~~ is ~~a~~the multifunctional NSs protein that is necessary for systemic movement in plants and can influence ~~alters~~ the emission of plant volatiles and suppress~~reduces~~ the JA-regulated plant defenses, resulting in the enhanced attractiveness of plants to flower thrips (*Frankliniella occidentalis*)~~,~~. NSs ~~also~~ can also functionally replace potyviral HC-Pro~~,~~ and promote systemic infection and symptom development by suppressing antiviral RNA silencing [6, 10–12]. The NSs ~~in~~protein of watermelon silver mottle virus (WSMoV) has the function of transmission of the virus by~~on~~ *T. palmi*~~.~~ ~~transmitted the virus~~ [13]. The NSs protein ~~in~~of TZSV~~,~~ ~~is an activator of~~activates a~~the~~ hypersensitive response in resistant plants and could ~~interacted~~interact with the VDAC protein ~~VDAC~~ in F. *ocicdentalis*, regulating the transmission of the persistent-propagative plant viruses [14]. The mechanisms of RNA-silencing suppression~~suppressor~~ in TZSV by NSs ~~in TZSV~~ and its role in virus infection ~~needed~~need further exploration.

Virus-induced gene silencing (VIGS) is a method to study~~determine~~ the ~~function~~functions of plant and pathogen genes by the agroinfiltration or biolistic inoculation of plants. VIGS has been successfully used to investigate gene function and disease resistance. Silencing of the *LeCTR1* gene in tomatoes led to an accumulation of ROS and increased the expression of *NPR1*, *PR1*, *PR5*, and *AOS2* ~~gene~~genes ~~expression~~ to prevent the ~~inhibit~~ *tomato leaf curl virus* (ToLCV) infection [15]. When the *H2B* and *CoiI* genes in *N. benthamiana* were silenced, the contents of the ~~phytohormonessalicylic~~ phytohormone salicylic acid (SA) and jasmonic acid (JA) increased, and the infection with potato virus X (PVX) and (TSWV) was inhibited [16, 17]. Macharia et al. found that silencing of the *NbHYPK* and *ATG8* genes could enhance ~~the~~ autophagocytosis ~~effect~~ and help ~~defend against~~combat the TMV infection [18]. ~~Qiu et al. inserted the~~The *N* gene of TSWV was inserted ~~*N* gene~~ into the~~a~~ TRV vector to further study the gene function, but there have been no studies on~~about~~ inserting TZSV genes into VIGS vectors [19]. In this study, *NSs* gene~~s~~ was silenced by constructing the~~onto~~ TRV-pTV00 vector and then infiltrated into *N. benthamiana* and *N. tabacum* cv. K326. RT-qPCR and ID-ELISA ~~analysis~~assays showed that *NSs* gene ~~replications~~ transcription and protein expression were inhibited more than 90.00%, and the

symptoms inof the silenced plants were silenced alleviated. We also observedshowed that the expression of the *Zingipain-2-like* gene significantsignificantly decreased when *NSs* genes was silenced. Furthermore, theThe results also showed that the co-localization expression of NSs-GFP and Zingipain-2-like-mCherrymCheery fusion protein could co-localization expression on the plasma membrane in *Nicotiana benthamiana* plants. This is the first report using theOur findings wea firstly used TRV VIGS system to analyze theanalysis functions of the TZSV gene function, whichand have important new implications for mechanistic studies of the silencing suppression of gene silencing by NSs and theirits effectseffect on virus systemic infection by the virus.

Materials and Methods

Materials

The pTV00, pBINTRA, and pTV00-PDS vectors were provided by the Professor Jianqiang Wu's's laboratory, at the Kunming Institute of Botany, Chinese Academy of Sciences (KIB CAS) [20]. The vector of pCAMBIA-GFP, pBI121-mCherry were obtained from our lab in the institute of biotechnology and germplasm resources. The TZSV YN-Chili isolate was collected from the infected tomato field tomato plants in Yuanmou, Yunnan Province, China, and was maintained onin *N. benthamiana* [1]. *N. tabacum* cv. K326 and *N. benthamiana* were cultivated at the Yunnan Academy of Agricultural Science. Primers were designed byusing the Primer 5 Design Program based onaccording to the sequences of the TZSV *NSs* genes and *Zingipain-2-like* gene published in the NCBI database (registration number: EF552433.1, LOC107763929) (Table S1).

TZSV inoculation

The frictionalfriction-based inoculation method was used to artificially infect *N. tabacum* cv. K326 and *N. benthamiana* plants at the 6 six-leaf stage. TZSV-infected *N. benthamiana* leaves were homogenized in the PBS buffer (100 mg/mL) (containing 137 mM NaCl, 1 mM KH_2PO_4, 8 mM$Na_2HPO_4 \cdot 12H_2O$, and 3 mM KCl), and there afterafter, which the homogenate was applied uniformly to 3 leaves per plant (1 mL). Ten minutes

after the TZSV inoculation, the inoculated plant leaves were rinsed with ddH$_2$O. ~~Plants~~Plant leaves inoculated with PBS buffer were used as controls, and five replicates were used for each sample. ~~After~~Five days after inoculation~~ for 5 days~~, ~~when~~ symptoms appeared~~, five replicates were used for each sample~~.

RNA extraction and RT-PCR

According to the instructions provided in~~of~~ the RNA extraction kit (Roche, America), the total RNA of~~ the~~ plant leaves was extracted. ~~A~~The first-strand cDNA synthesis kit was used for reverse transcription (TransGen, China). Q5 High-Fidelity DNA Polymerase (NEB, England) was used to amplify the target fragments. The PCR solution consisted of 1 μL of cDNA, 10 μL of 10× EasyTaq Buffer, 1 μL of 2.5 mM dNTPs, 2.5 μL of forward and~~primers, 2.5 μL of~~ reverse primers, and 0.5 μL of Q5 DNA polymerase; ddH$_2$O was added to the solution to obtain~~so that~~ the final volume of~~was~~ 50 μL. The PCR conditions were as follows: 35 cycles of denaturation at 98 °C for 40 s, 98 °C for 10 s, 60 °C for 20 s, and 72 °C for 30 s, followed by 72 °C for 2 min. The amplified products were subsequently analyzed~~imaged by~~using a UVP gel-imaging system.

Plasmid constructs

(i) ~~The fragments of the *NSs* gene to- be- silenced *NSs* gene were cloned in the~~into ~~pTV00 vectors.~~ PCR was used to amplify the desired fragments with specific primers (Table S1) using cDNA prepared from the plant tissues inoculated with TZSV. The target PCR fragments were excised, and ~~the~~ DNA was extracted ~~with~~using an appropriate kit (AxyGen, America). The ~~amplification~~amplified fragments of the *NSs* gene and ~~the~~ pTV00 vectors were digested by the restriction enzymes *BamHI* and *HindIII* according to the manufacturers' instructions, and the purified products of the *NSs* sequence were inserted into the pTRV-pTV00 vectors using T4 DNA ligase (NEB, England). The fragments of the to-be-silenced *NSs* gene were cloned in the pTV00 vectors. The vectors were then transformed into ~~*E. coli* DH5α~~ competent cells of *E. coli* strain DH5α. The selected positive clones were transferred~~sent~~ to Guangzhou Huada Gene Technology Co., Ltd., for sequencing. Plasmids with the correct sequence were

used to transform *A. tumefaciens* electrocompetent cells.

(ii) The sequences of *NSs* and *Zingipain-2-like* genes sequence was were amplified from the total RNA isolated from tobacco plants infected withby TZSV using reverse transcription- PCR (RT-PCR) and the special primers NSs-G-F/NSs-G-R (Table S1). The NSs-N and NSs-C PCR fFragments of *NSs* and *Zingipain-2-like* genes were digested with endonuclease Nco1 and Spe1 and inserted into vector of pCAMBIA-GFP and pBI121-mCherry using the same restriction sites to obtain pCAMBIA-NSs- N-GFP and pBI121-Zingipain-2-like-mCherrypCAMBIA-NSs-C-GFP, respectively. The Golgi markermarkers ST52-mCherry was amplified from the total RNA isolated from both tobacco and Arabidopsis [21].

Infiltration of the VIGs vector in Tobacco Leaves

The pTRV-pTV00-NSs vector constrcture was transformed into *Agrobacterium tumefaciens* strain GV3101 by electroporation. For every 1 mL of the inoculation solution, 2.5 mL of the liquid YEP culture plus 2.5 mL of pBINTRA liquid YEP culture waswere used. For each construct, athe final inoculation solution was made by mixing equal volumes of the resuspended *Agrobacterium* carrying the pTRV-pTV00-NSs construct and pBINTRA.

Inoculations wereInoculation of pTRV-pTV00-NSs construct was performed using a 1-mL syringe, and the inoculation solution of 1 mL was pressure injected into 3 individual leaves and per-plant was injected 3 leaves. –Phytoene dehydrogenase gene (*PDS*) as an indicator gene, the plant leaves will turn white when it was silenced, so the leaves injected with pTRV-pTV00-PDS construct becoming Bleaching bleached of the leaves onof the PDS-positive control occurred at approximately 10–14 days, the leaves performed with pTRV-pTV00-NSs construct post-inoculationinoculated, withand TZSV was inoculated. Plants injected with pTRV-pTV00 and plants inoculated with TZSV alone served as positive controls. At approximately 5–10 days post-inoculation, samples were collected, and healthy plants were used as blank controls.

Confocal laser scanning microscopy and co-localization assays

The leafLeaf epidermis waswere dissected from the areas of the agroinfiltrated

leaf area of N.*N.* *benthamiana* leaves and placedmounted in water between two coverslips. The confocalConfocal images were captured with thean inverted TCS SP8 and 10× water immersion objective lenses. GFP was excited at a wavelength ofwith 488 nm, wavelength and emissions were captured at 497–520 nm captured. Moreover, mCherry waswere excited atwith a wavelength of 561 nm wavelength and emissions were captured at 585–615 nm captured. Images were processed using athe TCS SP8 and Adobe (San Jose, CA, USA) Photoshop.

RT-qPCR

The sequences of the *NSs* gene were inserted into the pMD18-T vectorvectors, and the plasmids were diluted with a 10-fold continuouswith a gradient to obtain plasmids withat 10-fold dilution series concentration differences (10^{-1},10^{-2},10^{-3},10^{-4},10^{-5},10^{-6}), and then which were used as calibrators to construct a standard curve. The reaction system consisted of the following components: including 1 μL of cDNA, 2.5 μL of forward andprimers, 2.5 μL of reverse primers,1 0 μL of FastStart Universal SYBR Green Master (Rox), and 4 μL of ddH_2O. The reaction conditions were as follows: initial denaturation step at 95 °C for 10 min, followed by 40 cycles of 95 °C for 10 s, 57 °C for 30 s, and 72 °C for 30 s.

ID-ELISA

The contentscontent of the genes proteins werewas tested by ID-ELISA according to the antibody instructions for antibodies to determine the antiviral activity of the VIGS. Leaves (0.2 g) were homogenized usingwith a mortar and pestle and diluted 1: 3 in a 1/3 dilution with PBS buffer. Crude extracts (100 μL) were added intoto ELISA plate wells and incubated at 37 °C for 2 h. The plate was then washed with PBST buffer. TZSV NSs rabbit antibodies were diluted in a conjugationconjugate buffer. Afterward, and afterward, 100 μL of AP conjugated goat anti-rabbit IgG-AP conjugate (Sigma, USA) was added to each well. The color-developing solution was dissolved in p-nitrophenyl phosphate disodium hexahy-drate (Sigma-Aldrich) in substrate buffer to obtain a final concentration of 1 mg/mL. The absorbance was determined at 405 nm was measure dusing athe ELx 808 microplate ELISA reader (Bio-Tek, USA). Healthy

leaves were used as negative controls, PBS buffer was used as a blank control, and TZSV-infected leaves were used as positive controls. The PBS buffer was used as a blank control.

Results

NSs wasis localized to the PM and Golgi bodies

Previous studies have shown that confocal laser scanning microscopy (CLSM) wasalways used to analyzeanalysis the protein localized in living cells [22]. N.*N.* *benthamiana* is also a modelan ideal plant species in which to assess the subcellular localization of viral proteins. To characterize the subcellular targetingtarget of the NSs in plant cells, we first transiently expressed the recombinant NSs-GFP (green fluorescent protein) was first transiently expressed in leaf epidermal cells of N.*N.* *benthamiana* by agroinfiltration. We detected NSs-GFP was then detected to be associated within a structure that was the plasma membrane structures by CLSM (Fig 1 A-C). To determine whether the NSs-GFP bodies are colocalized with the Golgi stacks, we also checked the localization of NSs-GFP for Golgi bodies using the marker ST52-mCherry [23]. At 48 h afterAfter agroinfiltration onof N.*N.* *benthamiana* 48 h, we found that the NSs-GFP bodies are colocalized with the Golgi stacks (Fig 1 D-F), suggesting that the NSs-GFP protein was targetedtargets to the Golgi apparatus.

Silencing of the *NSs* gene

TRV vector wasis widely used to study the interactions between viruses and hosts and the functions of plant genes [24]. In the present study, Specificspecific primers (TZVNSsF1/TZVNSsR1) containing *BamHI* and *HindIII* restriction enzyme recognition sites were used to amplify the *NSs* gene fragments (Fig S1), and the DNA was inserted into athe pEASY-Blunt-Zero vector (TransGen, Beijing) for sequencing to ensure that the base sequences were not mutated.

The copies of the *NS*s gene were analyzedwas determined by RT-qPCR. The fragments of the *NSs* gene were amplified and inserted into thea pEASY-T1 Simple vector (Trans, Beijing). The concentrationconcentrations and the $OD_{260/280}$ value for the recombinant plasmid containing the *NSs* gene, were 225.82 ng/μL and 1.83,

respectively. The plasmid DNA with gradientGradient dilutions of (10^{-1} to 10^{-6}) of plasmid DNA was used as a templatetemplates. Standard curves and amplification curves of RT-qPCR data forof the *NSs* genesgene werewas obtained by automatic analysis performed by the software system— software. The standard curve equationequations of the *NSs* gene was Y =-3.444X+34.42, and the amplification efficiencyefficiencies were 90.90%, and the correlation coefficientcoefficients waswere 90.90% and 0.998, respectively. The results showed that the plasmid DNA could be used as a calibration product could be used to determinemeasure the copy numbers of the gene.

The *PDS* gene was used as a positive control to ensureindicate whether the success of silencing was successful. WithoutThe leaves of *N. benthamiana* without this gene in the plant, the *N. benthamiana* leaves were bleached. However, there were no phenotypic changes in the leaves of the *N. tabacum* cv. K326, despite the fact that thisthese species belongbelonging to the same genus. TZSV was inoculated into bothon the two species plants tobaccos that the plants were injected with pTRV-pTV00-NSs construct and positive control plants (inoculated only with TZSV), respectively, when the leaves occurred white in the veins of *N. benthamiana* leaves turned white injecting with pTRV-pTV00-PDS construct. TZSV was inoculated afterAtAfter 5 days afterof inoculation with TZSV, the the shrinkage appeared on the leaves of *N. benthamiana* plants injected with the pTRV-pTV00-NSs vector before inoculation with TZSV (Fig 2A). Ssevere leaf shrinkage also occurred in positive control plants (inoculated only with only TZSV) exhibited severe shrinkage symptoms (Fig 2B)., and The shrinkage also appeared on the leaves of *N. benthamiana* plants injected with the pTRV-pTV00-NSs vector before inoculation with TZSV began to display light shrinkage symptoms (Fig 2A),; however, for *N. tabacum* cv. K326, the symptoms were not different betweenin the VIGS plants and positive control plants were not different. To further determine the effects of the *NSs* gene, itsthe transcription expression levelof the *NSs* gene was measured by RT-qPCR. and found to be RT-qPCR analysis showed that the expression of the *NSs* gene significantly decreased compared to that of the positive control (inoculated only with only TZSV) in both hosts., The and the silencing efficienciesefficiency of the TZSV

NSs gene were~~was~~ 99.16%~~,~~ in *N. benthamiana* and 92.24% in *N. tabacum* cv. K326 (Fig 3A, B). ~~Taken together, the~~The results indicate~~indicating~~ that the pTRV-pTV00-NSs VIGS vector was successfully~~successful~~ constructed, and the *NSs* gene might be associated with TZSV infection.

Inhibition of the NSs protein expression

The NSs protein levels~~contents~~ ~~was~~were measured by ID-ELISA at 3~~ d~~, 5~~ d~~, 7~~d~~, and 9 ~~d~~days post-~~-~~inoculation with TZSV, ~~.~~ and found to be significantly decreased ~~The content of NSs protein~~ in leaves of *N. benthamiana* ~~leaves~~ and *N. tabacum* cv. K326 plants treated with the pTRV-pTV00-NSs ~~vector~~ construct and inoculated with TZSV ~~significantly decreased~~ compared to those~~that~~ in ~~the~~ positive plants at 7~~d~~ and 9 days~~d~~ (Table ~~2~~1). The results showed that the expression of the *NSs* protein in both *N. benthamiana* and *N. tabacum* cv. K326 hosts was inhibited.

The dependence of *NSs* gene silencing ~~depending~~ on the *zingipain-2-like* gene

The *Zingipain-2-like* gene was a homocysteine protease, which possesses cysteine-type endopeptidase activity and participates in the regulation of plant-type hypersensitive response [25, 26]. To confirm whether the host factor ~~of~~for the *Zingipain-2-like* gene interacts with RNA silencing suppressing~~suppressor~~ by the NSs gene of TZSV, the ~~replication~~ expression of the *Zingipain-2-like* gene performed by *NSs*, the silencing suppressor in plants, was detected by RT-qPCR assay, ~~that *NSs* gene silenced. The results showed that the replication of *Zingipain-2-like* gene~~ and found to be significantly decreased in the *NSs* gene-~~-~~silenced plants compared to ~~the~~ positive plants~~-~~, compared with the CK, expression of the *Zingipain-2-like* gene was up-regulated in both positive and NSs-silenced plants infected by TZSV plants, but higher in the positive plant. ~~*Zingipain*~~The rationale behind these data could be that *Zingipain-2-like* gene expression was induced by NSs (Fig 3 C).

To further ~~identify~~ investigate whether NSs and the Zingipain-2-like protein were co-localized and to identify the specific structures in living cells, we transiently expressed the recombinant NSs-GFP (green fluorescent protein) and Zingipain-2-like-

RFP (red fluorescent protein) in leaf epidermal cells of ~~N.~~*N.* *benthamiana* by agroinfiltration. ~~Co-expression~~ The co-expression of NSs-GFP with the Zingipain-2-like-RFP confirmed the co-localization of NSs and Zingipain-2-like ~~co-localized, and localization~~ at~~to~~ the plasma membrane by the confocal laser scanning microscope (CLSM) (Fig 4 A-C). Our results clarified~~clarify~~ that the RNA silencing suppressor ~~by~~ (NSs) ~~made~~ ~~closely~~had a close relationship with Zingipain-2-like when TZSV infected the plants.

Discussion

The importance of NSs for tospoviral infection in plants was first discovered ~~in plants~~ in the early 1990s.~~,~~ ~~a~~A higher virulence of TSWV isolates and more severe symptoms were~~was~~ observed ~~when~~with the elevated levels of NSs expression [27]. The TSWV NSs protein ~~which~~ acts as a suppressor of RNA silencing~~,~~ through binding small and long dsRNA and ~~suppressing~~ suppresses ~~local~~ short and long-distance systemic silencing to enhanced viral ~~us~~ accumulation and movement [28]. It also represents the Avr factor of the *Tsw* resistance gene in pepper [29, 30]. NSs of TSWV directly interact~~s~~ with *MYC2*, a regulator of the JA signaling, to disable JA-mediated activation of terpene synthase genes and ~~attenuates~~ attenuate host defenses, increasing~~increases~~ the attractiveness~~attraction~~ of the plants to thrips, ~~to~~and thus transmitting~~transmit~~ the disease [31]. So far, the ability of NSs of TSWV ~~NSs~~ to counteract defense mechanisms mediated by~~the~~ RNA silencing ~~defense mechanism~~ in plants has been demonstrated ~~was illuminated,~~; however, the mechanism of RNA silencing suppression by NSs in TZSV ~~NSs~~ and its role in virus infection are~~have~~ not ~~been~~yet clear ~~clearly~~. In the present study, the accumulation of the virus decreased and ~~the symptom~~symptoms were alleviated when the *NSs* gene was silenced~~,~~. The expression of the *Zingipain-2-like* gene was found to be significantly decreased in the *NSs* gene-silenced plants compared to positive plants, compared with the CK, expression of the Zingipain-2-like gene was up-regulated in both positive and NSs-silenced plants infected by TZSV plants, but higher in the positive plant.~~and we~~We first revealed that the *Zingipain-2-like* gene might be associated~~associate~~ with this function.

The ~~plant's~~ innate immune system of plants has~~contained~~ two different layers,~~:~~ including microbe-associated (MAMP) or pathogen-associated molecular pattern (PAMP)-triggered immunity (PTI) and effector-triggered immunity (ETI). PTI is mediated by the corresponding membrane-anchored pattern recognition receptors (PRRs) in~~of~~ plants, which serve as the first line of defense against the pathogen. Many plant viruses and their encoded proteins that could inhibit PTI ~~were reported~~, ~~such as~~ like ~~TSWV~~ the NSs protein of TSWV that suppressed the production of reactive oxygen species (ROS), have been reported ~~and PTI was inhibited~~ [30, 32, 33]. In this study, we found that NSs and Zingipain-2-like were co-localized at~~to~~ the plasma membrane; ~~and~~ the expression of the *Zingipain-2-like* gene significantly decreased in *NSs* gene-silenced plants compared to ~~the~~ positive plants, and Zingipain-2-like ~~take~~took part in the regulation of hypersensitive response, suggesting~~it suggested~~ that NSs and Zingipain-2-like might be associated with the activation of~~to activate~~ PTI-like responses.

In this study, ~~N.~~*N.* *benthamiana* was used as a model plant~~system,~~ and ~~the first time found~~ the ability of the NSs protein of TZSV to~~could~~ target ~~into~~ Golgi bodies in plant cells was observed for the first time. The targeting of the virus~~viral~~ glycoproteins to the Golgi apparatus plays a pivotal role in the formation of enveloped spherical particles of ~~for~~ the viruses belonging to~~in~~ the Bunyaviridae family [34–36]~~;~~. However, ~~Why~~the reason for the facilitation of ~~NSs protein facilitate~~ the formation of ~~an~~ enveloped spherical particles~~particle~~ by the NSs protein remains to be extensively investigated ~~in the future~~.

In this study, TRV vectors were used to construct VIGS vectors of the *NSs* gene ~~to~~for the analysis of their ~~the~~ functions~~function~~. The results of RT-qPCR, ~~and~~as well as the plant disease symptoms, showed that the gene replication was inhibited ~~to~~ up to 90%. ID-ELISA showed that the protein contents also significantly decreased. The high efficiency of gene silencing can be verified~~measured~~ by sampling and testing immediately at~~when~~ the onset of the disease ~~appears on the plants~~. At the same time, temperature also had~~has~~ effects on the silencing phenotypes in~~of~~ plants [37]. In this experiment, the temperature was strictly controlled~~;~~, and thus, the gene was silenced at

relatively high levels, and the duration was relatively long. TRV has a wide range of hosts, withand there is a significant difference in sensitivity to TRV between species and cultivars [38, 39]. For instance, TRV sensitivity testing was carried out on 21 Gerbera gerbera cultivars, and the results revealed that only 5 cultivars showed photo bleached PDS silencing symptoms on newly developed leaves [40]. In this study, the *N. benthamiana* leaves were bleached, but there were no phenotypic changes in the leaves of *N. tabacum* cv. K326, despite the fact that both species belongbelonging to the same genus, indicating that the TRV-VIGS vector exhibited differentdiffering sensitivities to different host species. TRV has a wide range of hosts, with a significant difference in sensitivity to TRV between species and cultivars [38, 39]. For instance, TRV sensitivity testing was carried out on 21 gerbera cultivars, and the results revealed that only 5 cultivars showed photobleached PDS-silencing symptoms on newly developed leaves [40]. The VIGS method can be used for reverse genetics studiesresearch and the analysis ofto determine the functions of unknown genes.

In summary, our results presented here revealed that NSs, a suppressor of RNA silencing NSs ofin TZSV, was localized to the PM and Golgi bodies and might be alsoalso be associated with Zingipain-2-like to activate PTI-like responses taking advantage ofusing VIGS and subcellular localization predictionmethods.

Acknowledgements

The authors would like to thank Professor Jianqiang Wu's laboratory KIB. CAS. for providing the VIGS vectors. We also thank the Yunnan Academy of Agricultural Sciences Yunnan Provincial Key Lab of Agricultural Biotechnology for equipment support of the confocal microscope.

Supporting information

S1Table. Special primers. The primers were used to construct the VIGS, fluorescence labeling, and RT-qPCR vectors.

S1 File. Amplication of the *NSs* gene sequence for VIGs.

Formatted: Font: (Default) Times New Roman, Bold

Author Contributions

Conceptualization: Li-Hua Zhao.

Formal analysis: Zhong-Kai Zhang.

Methodology: Ting-Ting Li, Si-Chen, Yue Chen, Run-Shuang Qiu

Resources: Li-Hua Zhao, Zhong-Kai Zhang.

Supervision: Li-Hua Zhao

Validation: ZhongKai-Zhang.

Writing – original draft: Jing-Li, Si-Chen, Li-Hua Zhao.

Writing – review & editing: Jing-Li, Li-Hua Zhao, Li-Zhen Zhang, Xue Zheng, Zhong-Kai Zhang.

Funding

This study was supported by National Natural Science Foundation of China (No. U1802235, 31960531), Yunnan Fundamental Research Projects (202101AT070269).

Reference

1. Dong JH, ChengXF, Yin YY,Fang Q, Ding M, Li TT, Zhang LZ, Su XX, McBeath JH, Zhang ZK. Characterization of tomato zonate spot virus, a new *tospovirus* in China. Arch Virol. 2008; 153(5): 855-864.

2. Liu Y, Huang CJ, Tao XR, Yu HQ. First report of tomato zonate spot virus in *Iris tectorum* in China. Plant dis. 2015; 99(1): 164-164.

3. Zhu M, Jiang L, Bai BH, Zhao WY, Chen XJ, Li J, Yong Liu 2, Chen ZQ, Wang BT, Wang CL, Wu Q, Shen QH, Dinesh-Kumar SP, Tao XR. The intracellular immune receptor Sw-5b confers broad-spectrum resistance to Tospoviruses through recognition of a conserved 21-amino-acid viral effector epitope. Plant Cell. 2017; 29(9): 2214-2232.~~Wu XD, Wu XY, Li WB, Cheng XF. Molecular characterization of a divergent strain of calla lily chlorotic spot virus infecting celtuce (*Lactuca sativa* var. augustana) in China. Arch virol. 2018; 163(5): 1375-1378.~~

4. Cai JH, Qin BX, Wei X P, Huang J, Zhou WL, Lin BS, Yao M, Hu ZZ, Feng ZK, Tao XR. Molecular identification and characterization of tomato zonate spot virus in tobacco in Guangxi, China. Plant dis. 2011; 95(11): 1483-1483.

5. Niu YB, Wang DF, Cui LY, Wang BX, Pang XJ, Yu PX. Monoclonal antibody-based colloid gold immunochromatographic strip for the rapid detection of tomato zonate spot tospovirus. Virol J. 2018; 15(1): 15.

6. Takeda A, Sugiyama K, Nagano H, Mori M, Kaido M, Mise K, Tsuda S, Okuno T. Identification

of a novel RNA silencing suppressor, NSs protein of tomato spotted wilt virus. FEBS Lett. 2002; 532(1-2): 75-79.

7. Zhang ZK, Zheng KY, Dong JH,Fang Q, Hong J, Wang XF. Clustering and cellular distribution characteristics of virus particles of tomato spotted wilt virus and tomato zonate spot virus in different plant hosts. Virol. J. 2016; 13(1): 11.

8. Margaria P, Miozzi L, Rosa C, Axtell MJ, Pappu HR, Turina M. Small RNA profiles of wild-type and silencing suppressor-deficient tomato spotted wilt virus infected *Nicotiana benthamiana*. Virus Res. 2015; 208: 30-8.

9. Schnettler E, Hemmes H, Huismann R, Goldbach R, Prins M, Kormelink R. Diverging affinity of tospovirus RNA silencing suppressor proteins, NSs, for various RNA duplex molecules. J Virol. 2010; 84(21): 11542-54.

10. Margaria P, Bosco L, Vallino M, Ciuffo M, Mautino GC, Tavella L, Turina M. The NSs protein of tomato spotted wilt virus is required for persistent infection and transmission by *Frankliniella occidentalis*. J. Virol. 2014; 88: 5788-5802.

11. Garcia-Ruiz H, Sergio M, Peralta G, Patricia A. Maxwell H. Tomato spotted wilt virus NSs protein supports infection and systemic movement of a potyvirus and is a symptom determinant. Viruses. 2018; 10: 129.

12. Du J, Song XY, Shi XB, Tang X, Chen JB, Zhang ZH, Chen G, Zhang Z, Zhou XG, Liu Y, Zhang DY. NSs, the silencing suppressor of tomato spotted wilt orthotospovirus, interferes with JA-regulated host terpenoids expression to attract *Frankliniella occidentalis*. Frontiers in Microbiology. 2020; 11: 590451.

13. Mou DF, Chen WT, Li WH, Chen TC, Tseng CH, Huang LH, Peng JC, Yeh SD, Tsai CW. Transmission mode of watermelon silver mottle virus by Thrips palmi. PloS ONE. 2021; 16(3): e0247500.

14. Zhao XY, Chen JB, Wang SG, Zhang XL, Mu Y, Wei H, Zhao LH, Chen YD, Zheng X, Chen Y, Zheng LM. Zhang J. Screening and identification of the protein interacting with the NSs protein of tomato zonate spot virus in *Franiklinella ocicdentalis* (Thysanoptera: Thripidae) by yeast two-hybrid technique. Acta Entomologica Sinica. 2020; 63(6): 735-743. (in Chinese)

15. Chandan RK, Singh AK, Patel S, Swain DM, Tuteja N, Jha G. Silencing of tomato *CTR1* provides enhanced tolerance against tomato leaf curl virus infection. Plant Signal Behav. 2019;

14(3): e1565595.

16. Yang X, Lu YW, Zhao X, Jiang LL, Xu SC, Peng JJ, Zheng HY, Lin L, Wu YH, MacFarlane S, Chen JP, Yan F. Downregulation of nuclear protein H2B induces salicylic acid mediated defense against PVX infection in *Nicotiana benthamiana*. Front Microbiol. 2019; 10: 1000.
17. Zhao LH, Hu ZH, Li SL, Zhou XP, Li J, Su XX, Zhang LZ, Zhang ZK, Dong JH. Diterpenoid compounds from *Wedelia trilobata* induce resistance to tomato spotted wilt virus via the JA signal pathway in tobacco plants. Sci Rep. 2019; 9: 2763.
18. Macharia MW, Wilfred YZ, Das PP, Naqvi NI, Wong SM. Proximity-dependent biotinylation screening identifies *NbHYPK* as a novel interacting partner of *ATG8* in plants. BMC Plant Biol. 2019; 19: 326.
19. Qiu RS, Zhao LH, Zhang XM, Zhang J,Zheng X, Li J, Zhang ZK. Construction of a VIGS vector containing *N* gene derived from tomato spotted wilt orthospovirus. Acta phytopathologicasinica. 2020; 50(6): 766-733.
20. Ratcliff F, Martin-Hernandez AM, Baulcombe DC. Tobacco rattle virus as a vector for analysis of gene function by silencing. Plant J. 2001; 25(2): 237-245.
21. Dupas SJC, Nebenfu A, Boulaflous A, Gueye MLF, Plasson C, Hawes C, Driouich A, Faye L, Gomord V. Plant N-glycan processing enzymes employ different targeting mechanisms for their spatial arrangement along the secretory pathway. The Plant Cell. 2006; 18: 3182-3200.
22. Feng ZK, Xue F, Xu M, Chen XJ, Zhao WY, Garcia-Murria MJ, Mingarro I, Liu Y, Huang Y, Jiang L, Zhu M, Tao XR. The ER-membrane transport system is critical for intercellular trafficking of the NSm movement protein and tomato spotted wilt tospovirus. PLoS Pathog. 2016; 12(2): e1005443.
23. Nelson BK, Cai X, Nebenfuhr A. A multicolored set of in vivo organelle markers for colocalization studies in *Arabidopsis* and other plants. Plant J. 2007; 51: 1126-1136.
24. Tavares-Esashika ML, Campos RNS, Blawid R, da Luz LL, Inoue-Nagata AK, Nagata T. Characterization of an infectious clone of pepper ringspot virus and its use as a viral vector. Arch Virol. 2020; 165(2): 367-375.
25. Khanna-Chopra R, Srivalli B, Ahlawat YS. Drought induces many forms of cysteine proteases not observed during natural senescence. Biochemical & Biophysical Research Communications. 1999; 255(2): 324.

26. Rungsaeng P, Sangvanich P, Karnchanatat A. Zingipain, a ginger protease with acetylcholinesterase inhibitory activity. Appl Biochem Biotechnol. 2013; 170: 934-950.

27. Kormelink R, Kitajima EW, De Haan P, Zuidema D, Peters D, Goldbach R. The nonstructural protein (NSs) encoded by the ambisense S RNA segment of tomato spotted wilt virus is associated with fibrous structures in infected plant cells. Virology. 1991; 181: 459–468.

28. Hedil M, Kormelink R. Viral RNA silencing suppression: the enigma of Bunyavirus NSs proteins. Viruses. 2016; 8: 208.

29. De Ronde D, Pasquier A, Ying S, Butterbach P, Lohuis D, Kormelink R. Analysis of tomato spotted wilt virus NSs protein indicates the importance of the N-terminal domain for avirulence and RNA silencing suppression. Mol. Plant Pathol. 2014; 15: 185-195.

30. Huang C, Liu Y, Yu HQ, Yuan C, Zeng JM, Zhao L, Tong ZJ, Tao XR. Non-structural protein NSm of tomato spotted wilt virus is an avirulence factor recognized by resistance genes of tobacco and tomato via different elicitor active sites. Viruses. 2018; 10: 660.

31. Wu XJ, Xu SH, Zhao PZ, Zhang X, Yao XM, Sun YM, Fang RX, Ye Jian. The *Orthotospovirus* nonstructural protein NSs suppresses plant MYC-regulated jasmonate signaling leading to enhanced vector attraction and performance. PLoS Pathog 2019; 15(6): e1007897.

32. Hogenhout SA, Van der Hoorn RAL, Terauchi R, Kamoun S. Emerging concepts in effector biology of plant-associated organisms. Mol. Plant Microbe Interact. 2009; 22: 115-122.

33. Gupta R, Min CW, Kim SW, Yoo JS, Moon AR, Shin AY, Kwon SY, Kim ST. A TMT-based quantitative proteome analysis to elucidate the TSWV induced signaling cascade in susceptible and resistant cultivars of *Solanum lycopersicum*. Plants (Basel) 2020; 9(3): 290.~~Kamoun, S. Groovy times: Filamentous pathogen effectors revealed. Curr. Opin. Plant Biol. 2007; 10: 358-365.~~

34. Kikkert M, Van LJ, Storms M, Bodegom P, Kormelink R, Goldbach R. Tomato spotted wilt virus particle morphogenesis in plant cells. J. Virol. 1999; 73: 2288-2297.

35. Shi X, Lappin DF, Elliott RM. Mapping the Golgi targeting and retention signal of *Bunyamwera* virus glycoproteins. J. Virol. 2004; 78: 10793-10802.

36. Yao M, Liu XF, Li S, Xu Y, Zhou YJ, Zhou XP, Tao XR. Rice Stripe Tenuivirus NSvc2 Glycoproteins targeted to the Golgi body by the N-Termina transmembrane domain and adjacent cytosolic 24 amino acids via the COP I- and COP II-dependent secretion pathway.

Journal of Virology. 2014; 88(6): 3223-3234.

37. Fu DQ, Zhu BZ, ZhuHL, Zhang HX, Xie YH, Jiang WB, Zhao XD, Luo KB. Enhancement of virus induced gene silencing in tomato by low temperature and low humidity. Mol Cells 2006; 21(1): 153-160.

38. Bennypaul HS, Mutti JS, Rustgi S, Kulvinder SG. Virus induced gene silencing (VIGS) of genes expressed in root, leaf, and meiotic tissues of wheat. Funct Integr Genomics. 2012; 12 (1): 143-156.

39. Senthilkumar M, Mysore KS. Tobacco rattle virus-based virus-induced gene silencing in *Nicotiana benthamiana*. Nat Protoc. 2014; 9 (7): 1549-1562.

40. Deng X, Elomaa P, Nguyen CX, Hytönen T, Valkonen JPT, Teeri TH. Virus induced gene silencing for Asteraceae a reverse genetics approach for functional genomics in *Gerbera hybrida*. Plant Biotech. J. 2012; 10(8): 970-97.

Table 1 Detection of NSs protein content ~~after VIGS~~with different treatment in both host ~~by~~ using ID-ELISA

	pTRV-pTV00-NSs+TZSV				TZSV				pTV00+ TZSV				CK			
Host species	3 d	5 d	7 d	9 d	3 d	5 d	7 d	9 d	3 d	5 d	7 d	9 d	3 d	5 d	7 d	9 d
N. benthamiana	0.207 ± 0.010a	0.214 ± 0.055a	0.244 ± 0.019a	0.201 ± 0.011a	0.219 ± 0.001ac	0.289 ± 0.002ac	0.446 ± 0.008bc	0.369 ± 0.014bc	0.208 ± 0.010ac	0.287 ± 0.005ac	0.396 ± 0.002bc	0.379 ± 0.017bc	0.169 ± 0.009d	0.163 ± 0.013d	0.168 ± 0.014d	0.162 ± 0.013d
N. tabacum cv. K326	0.181 ± 0.009a	0.218 ± 0.007a	0.257 ± 0.006a	0.225 ± 0.062a	0.218 ± 0.006ac	0.282 ± 0.008ac	0.434 ± 0.043bc	0.391 ± 0.011bc	0.217 ± 0.007ac	0.245 ± 0.002ac	0.374 ± 0.010bc	0.368 ± 0.001bc	0.163 ± 0.006d	0.147 ± 0.007d	0.163 ± 0.004d	0.133 ± 0.008d

pTRV-pTV00-NSs +TZSV: ~~means~~ infiltrated pTRV-pTV00-NSs ~~VIGS vector~~construct and inoculated with TZSV; TZSV: positive control and means only inoculated with TZSV; pTV00-TZSV: means infiltrated pTRV-pTV00 ~~VIGS~~ vector and inoculated TZSV; CK: negative control and means the leaves with no treatment. All values are means ± SE. Means in a column followed by different letters are significantly different at $P \leq 0.05$.

Fig 1 The NSs protein in TZSV ~~NSs~~ is localized ~~with~~ at the PM and Golgi bodies

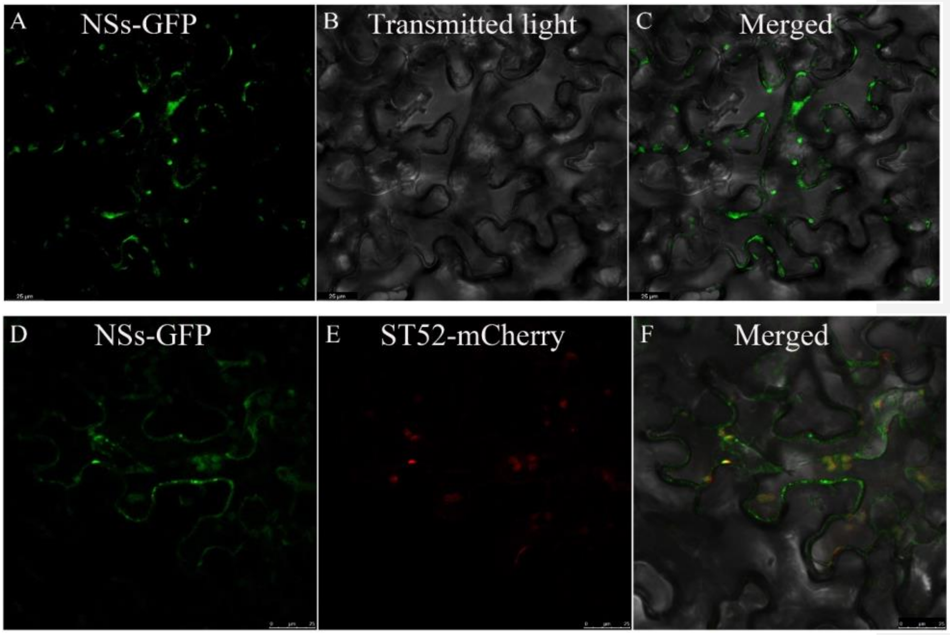

A-C: localization of NSs-GFP at 48 h post infiltration (hpi). Bar, 25 μm. D-F: ~~Co~~co-localization of NSs-GFP with Golgi bodies at 48 hpi. Bar, 25 μm. The fluorescence derived from *N. benthamiana*

leaves was monitored using a confocal Leica TCS SP8.

Fig 2 The symptom~~s~~ of TZSV infection on *N. benthamiana* ~~after *NSs* gene silenced~~

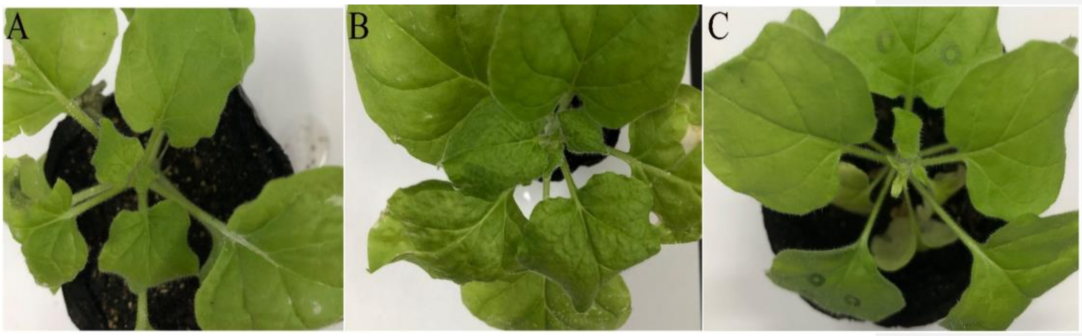

A: infiltrating pTRV-PTV00-NSs construct in the ~~plant of~~ leaves ~~infiltrating pTRV-PTV00-NSs vector firstly~~ and inoculated with TZSV; B: posivite control ~~of the plant~~ (inoculated with TZSV only); C: negative control(the healthy plant)

Fig 3 RT-qPCR detected t~~T~~he ~~replication~~ transcription of *NSs* and *Zingipain-2-like* genes after *NSs* gene silenced ~~detected by RT-qPCR~~

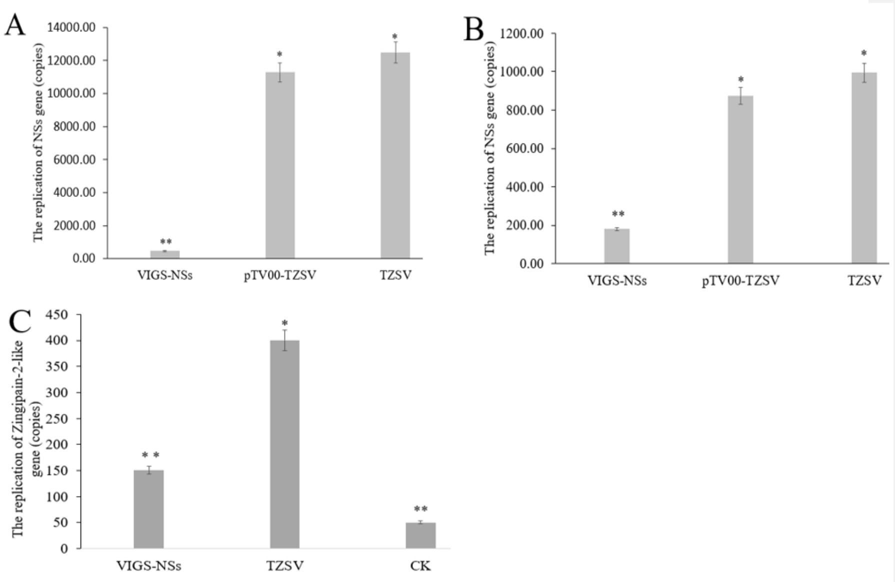

A: the expression of *NSs* gene is detection detected with different treatment of *NSs* gene expression after VIGS and inoculated TZSV at 5d in *Nicotiana Benthamiana* leaves; B: the expression of *NSs* gene is detected with different treatment detection of *NS* gene expression after VIGS and inoculated TZSV at 5d in *N. tabacum* cv. K326 leaves; C: the expression detection of *Zingipain-2-like* gene is detected expression after VIGS and inoculated TZSV at 5d in *Nicotiana Benthamiana* leaves; VIGS-NSs: infiltrated pTRV-pTV00-NSs VIGS vectorconstruct and inoculated with TZSV; pTV00-TZSV: infiltrated pTRV-pTV00 VIGS vector and inoculated with TZSV; TZSV: positive control and only inoculated with TZSV; CK: negative control (the healthy plant). All values are means ± SE. *means differences are significantly different at $P \le 0.05$.

Fig 4 The NSs protein in TZSV NSs is co-localized with the Zingipain-2-like protein

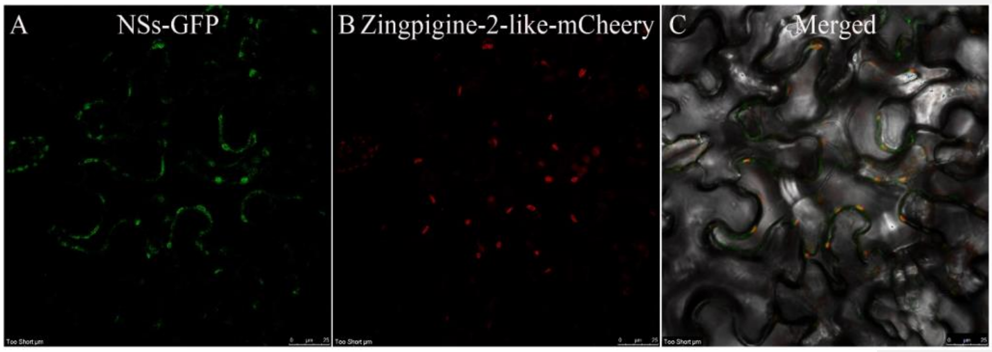

A-C: ~~Co~~co-localization of NSs-GFP with Zingipain-2-like at 48 hpi. Bar, 25 μm. The fluorescence derived from *N. benthamiana* leaves was monitored using a confocal Leica TCS SP8.

Answer to reviews

Review 1

1. Virus taxonomy and writing of scientific name and virus name. TZSV should belong to Tomato zonate spot orthotospovirus species, Orthotospovirus genus, Tospoviridae family, Bunyavirales order. Authors can visit the ICTV website for information. For abbreviation, a virus name is required. Therefore, the use of 'tomato zonate spot virus (TZSV)' is recommended.

Answer: I have changed "Tomato zonate spot orthotospovirus" into "tomato zonate spot virus" in the article.

2. The text needs to be completely corrected. Too many spelling and grammatical errors can be found in the text. In addition, the citation of some references seems inappropriate.

Answer: I have completely corrected the text and documented "changed point by point"; some references have been changed, including: Page 12, line 317-319: the reference "Wu XD, Wu XY, Li WB, Cheng XF. Molecular characterization of a divergent strain of calla lily chlorotic spot virus infecting celtuce (Lactuca sativa var. augustana) in China. Arch virol. 2018; 163(5): 1375-1378." has been changed into "Zhu M, Jiang L, Bai BH, Zhao WY, Chen XJ, Li J, Yong Liu 2, Chen ZQ, Wang BT, Wang CL, Wu Q, Shen QH, Dinesh-Kumar SP, Tao XR. The intracellular immune receptor Sw-5b confers broad-spectrum resistance to Tospoviruses through recognition of a conserved 21-amino-acid viral effector epitope. Plant Cell. 2017; 29(9): 2214-2232."Page 15, line 405-406: the reference "Kamoun, S. Groovy times: Filamentous pathogen effectors revealed. Curr. Opin. Plant Biol. 2007; 10: 358-365." has been changed into "Gupta R, Min CW, Kim SW, Yoo JS, Moon AR, Shin AY, Kwon SY, Kim ST. A TMT-based quantitative proteome analysis to elucidate the TSWV induced signaling cascade in susceptible and resistant cultivars of *Solanum lycopersicum*. Plants (Basel) 2020; 9(3): 290."

3. The legends of tables and figures have to be clearly described for readers to understand. Additionally, miscitation of tables and figures can be found. For example, the primers are listed in Table S1, but the citation is in Table 1 (page 4, line 93, and page 5, line 129); Table 2 is quoted at page 9 (line 229), there is actually no Table 2!

Answer: I have described the legends of tables and figures again in the "changed point by point file"; the citation is in Table 1 (page 4, line 93, and page 5, line 129) has been changed into "Table S1"; Table 2 is quoted at page 9 (line 229) has been changed into "Table 1".

4. What is 'PDS', 'TCS SP8', 'PM', etc.? When describing an acronym for the first time, the full name must be provided.

Answer: the full name was added when describing an acronym for the first time, for example, Phytoene dehydrogenase gene (*PDS*); insert the word "(PM)" followed the word membrane, confocal laser scanning microscope (CLSM); the acronym 'TCS SP8'is the machine model.

5. The results, such as the quantification of NSs gene (standard curve and copy number, page 8, lines197-206) and the phenomenon and molecular evidence of PDS gene silencing in the tested plants (lines 207-223), should be illustrated in figures. Was PDS silencing suppressed by the TZSV NSs protein? It is not clear about the response to TZSV infection (or NSs protein) in the PDS-silenced tobacco plants! What is the point of this? In fact, I am confused about Fig. 2. I think the PDS silencing here is meaningless!

Answer: PDS silencing suppressed was not by the TZSV NSs protein, *PDS* as an indicator gene, the plant leaves will turn white when it was silenced that the leaves injected with pTRV-pTV00-PDS construct, the *NSs* gene might be silenced that the leaves injected the pTRV-pTV00-NSs construct.

Please explain why PDS silencing is not meaningless.

6. Authors must explain why the Zingipain-2-like gene is investigated? It is not even described in the M & M section! Logically, preliminary research should be performed to reveal the possible role of Zingipain-2-like gene in orthotospoviral (or TZSV) infection.

Answer: the section "*NSs* gene sequence was amplified from total RNA isolated from tobacco infected by TZSV using reverse transcription PCR (RT-PCR) and the primers

NSs-G-F/ NSs-G-R (Table 1). The NSs-N and NSs-C PCR Fragments were digested with Nco1and Spe1 and inserted into pCAMBIA-GFP using the same restriction sites to obtain pCAMBIA-NSs-N-GFP and pCAMBIA-NSs-C-GFP, respectively. The Golgi markers ST52-mCherry was amplified from total RNA isolated from tobacco Arabidopsis [21]" has been changed into "The sequences of *NSs* and *Zingipain-2-like* genes were amplified from the total RNA isolated from tobacco plants infected with TZSV using reverse transcription-PCR (RT-PCR) and the special primers (Table S1). The PCR fragments of *NSs* and *Zingipain-2-like* genes were digested with endonuclease and inserted into vector of pCAMBIA-GFP and pBI121-mCherry to obtain pCAMBIA-NSs -GFP and pBI121-Zingipain-2-like-mCherry, respectively. The Golgi marker ST52-mCherry was amplified from the total RNA isolated from both tobacco and Arabidopsis [21]."

Where is your answer to the yellow highlight?

7. The description of 'replication' of NSs gene or Zingipain-2-like gene is incorrect. It is should be 'transcription'!

Answer: I have changed the word "replication" into "transcription" in the paper. For example: page 1, line 20: the word "replications" has been changed into "transcription"; Page 3, line 74: the word "replications" has been changed into "transcription".

Reviewer #2: The manuscript (PONE-D-21-26999) describes the functional investigation of NSs gene of tomato zonate spot orthotospovirus. The authors showed that NSs protein (fused with GFP) localized in plasma membrane and Golgi bodies. They then constructed NSs-silenced tobacco plants by VIGS and then inoculated with tomato zonate spot orthotospovirus (TZSV). By monitoring the symptom expression and detection the NSs expression level, mild symptom was observed in N. benthamiana, but not in N. tabacum cv. K326. However, NSs was decreased by more than 90% in both plants. They also found that the expression of a host gene, Zingipain-2-like gene, seemed to be induced by the expression of NSs, and these two proteins co-localized in the cell. This was the first demonstration of the involvement of NSs in infection of TZSV. While the story is of interest, flaws need to be fixed before acceptance for publication.

1. Title: changed to “Functional analysis of the nonstructural protein NSs of tomato zonate spot orthotospovirus”

Answer: the title has been changed into “Functional analysis of the nonstructural protein NSs of tomato zonate spot virus”.

2. Abstract: needs to be rewritten after modification of the text.

Answer: the Abstract “Tomato zonate spot orthotospovirus (TZSV), a member of the genus *orthotospovirsus*, causing severe reductions of vegetables and ornamental crops in southwest China. The NSs protein is an RNA silencing suppressor in *orthotospovirsus*, such as TZSV, but the mechanism of NSs and its role in virus infection are poorly understood. Here, we showed that an NSs-GFP fusion protein transiently expressed on plasma membrane and the Golgi bodies in *Nicotiana benthamiana* plants. When TZSV *NSs* gene was silenced and infiltrated into *N. benthamiana* and *N. tabacum* cv. K326. RT-qPCR and Indirect enzyme-linked immunosorbent assay (ID-ELISA) analysis showed that *NSs* gene replications and protein expression were inhibited more than 90.00%, and the symptoms of the plants silenced alleviated. We also showed that the expression of *Zingipain-2-like* gene significant decreased when *NSs* genes was silenced, the NSs-GFP and Zingipain-2-like-mCheery fusion protein could co-localization expression. The results of this study provide new insight into the mechanism of silencing suppression by NSs and its effect on virus systemic infection, and support the theory of resistance breeding and preventing and controlling TZSV in the field.” has been changed into “Tomato zonate spot virus (TZSV), a member of the genus *orthotospovirsus*, causes severe damage to vegetables and ornamental crops in southwest China. The NSs protein is an RNA silencing suppressor in various *orthotospovirsus* like TZSV, but its mechanism and role in virus infection are poorly understood. Here, we observed that an NSs-GFP fusion protein was transiently expressed on the plasma membrane and Golgi bodies in *Nicotiana benthamiana* plants. The TZSV *NSs* gene was silenced and infiltrated into *N. benthamiana* and *N. tabacum* cv. K326. RT-qPCR and Indirect enzyme-linked immunosorbent assay (ID-ELISA) showed that the replications and the protein expression of the *NSs* gene were inhibited by more than 90.00%, and the symptoms on

silenced plants were alleviated. We also found that the expression of the *Zingipain-2-like* gene significantly decreased when the *NSs* gene was silenced, resulting in co-localization of the NSs-GFP and the Zingipain-2-like-mCherry fusion protein. The findings of this study provide new insights into the mechanism of silencing suppression by NSs, as well as its effect on systemic virus infection, and also support the theory of disease resistance breeding and control and prevention of TZSV in the field." .

3. Materials and Methods: materials and methods should be introduced in a logical manner, e.g., NSs gene amplification should go ahead of the construction of NSs gene silencing construct.

Answer: the sentence "The fragments of the to-be-silenced *NSs* gene were cloned in the pTV00 vectors." has been transferred to the location before the sentence "The vectors were then transformed into *E. coli* DH5α competent cells.".

1) pCAMBIA-GFP, where was it obtained?

Answer: The vector of pCAMBIA-GFP was obtained from our lab in the institute of biotechnology and germplasm resources, and insert this sentence before the sentence "The TZSV YN-Chili isolate was collected from infected field tomato plants in Yuanmou".

The reviewer asked where was pCAMBIA-GFP originally obtained from? Please also indicate where in the revised ms such information is provided.

2) Vector and construct are different. pTRV-pTV00-NSs is a construct, not a vector.

Answer: page 5, line 131: the words "pTRV-pTV00-NSs -vector" have been changed into "pTRV-pTV00-NSs -construct";

3) Amount of inoculum used in leaf infiltration?

Answer: Inoculation was performed using a 1-mL syringe, and the inoculation solution of 1 mL was pressure injected into individual leave and per plant was injected 3 leaves.

Where is this information in the revised ms?

4) Lines 141-143: Bleaching of the leaves of the PDS-positive control occurred at approximately 10-14 days post-inoculation, and TZSV was inoculated. — what did the authors mean?

Answer: Phytoene dehydrogenase gene (*PDS*) as an indicator gene, the plant leaves will turn white when it was silenced, so the leaves injected with PDS vector becoming bleaching at approximately 10–14 days, the leaves performed with pTRV-pTV00-NSs construct inoculated with TZSV.

Where is the information in the revised ms?

5) Line1 164-165: The content of the genes was tested by ID-ELISA according to the antibody instructions to determine the antiviral activity of the VIGS. — not genes but proteins?

Answer: line 159-160: the sentence "The content of the genes was tested by ID-ELISA according to the antibody instructions to determine the antiviral activity of the VIGS." has been changed into "The contents of the proteins were tested by ID-ELISA according to the instructions for antibodies to determine the antiviral activity of VIGS.".

4. Results

1) Line 197: The copies of NSs gene was determined by RT-qPCR. — for what purpose?

Answer: The silencing efficiencies of the TZSV *NSs* gene were determined and the results indicate that the pTRV-pTV00-NSs VIGS vector was successfully constructed, TZSV infection was prevented due to the *NSs* gene might be associated with TZSV infection. Your reply did not answer the question.

2) Line 212-217: the sentence needs to be reorganized, Fig.2A goes before Fig. 2B

Answer: Page 8, line 206-209: the sentence "TZSV was inoculated after 5 days, the positive control (inoculated with only TZSV) exhibited severe shrinkage symptoms (Fig 2B), and the leaves of *N. benthamiana* plants injected with the pTRV-pTV00-NSs vector before inoculation with TZSV began to display light shrinkage symptoms (Fig 2A)," has been changed into "After 5 days of inoculation with TZSV, the shrinkage appeared on the leaves of *N. benthamiana* plants injected with the pTRV-pTV00-NSs vector before inoculation with TZSV (Fig 2A). Severe leaf shrinkage also occurred in positive control plants (inoculated only with TZSV) (Fig 2B);".

3) Line 212: TZSV was inoculated after 5 days,…--- compared with line 142 "10-14 days"?

Answer: two different assays. Firstly, Phytoene dehydrogenase gene (*PDS*) as an indicator gene, the plant leaves will turn white when it was silenced, so the leaves injected with PDS vector becoming bleaching at approximately 10–14 days; secondly, the leaves performed with pTRV-pTV00-NSs construct inoculated with TZSV. After 5 days of inoculation with TZSV, the shrinkage appeared on the leaves of *N. benthamiana* plants injected with the pTRV-pTV00-NSs vector before inoculation with TZSV (Fig

2A). Severe leaf shrinkage also occurred in positive control plants (inoculated only with TZSV) (Fig 2B).

4) Line 235: “the replication of Zingipain-2-like gene…”, changed to expression of Zingipain-2-like gene. Also the title of Fig. 3 should be changed accordingly.

Answer: Page 8, Line 235: the word “replication” has been changed into “expression”; Page 17, line 455: the title “The replication of *NSs* and *Zingipain-2-like* genes detected by RT-qPCR” has been changed into “RT-qPCR detected the transcription of *NSs* and *Zingipain-2-like* genes after *NSs* gene silenced”.

5) Line 234-238: Fig. 3C does not tell whether or not NSs interacts with Zingipain-2-like gene. The fact was that Zingipain-2-like gene expression was induced by NSs. Data in Fig. 3C was inaccurately explained in the text, compared with the CK, expression of the Zingipain-2-like gene was up-regulated in both normal and NSs-silenced plants infected by TZSV, but higher in the normal plant (positive plant). The rationale behind these data could be that the NSs in the positive plant was higher than in the NSs-silenced plant.

Answer: Page 8, line 227: insert the words “the regulation of” followed the word “in”. Page 8-9, line 228-232: the section “To confirm whether the host factor of *Zingipain-2-like* gene interact with RNA silencing suppressor by NSs of TZSV, the replication of *Zingipain-2-like* gene in plants was detected by RT-qPCR that *NSs* gene silenced. The results showed that the replication of *Zingipain-2-like* gene significantly decreased in *NSs* gene silenced plants compared to the positive plants (Fig 3 C).” has been changed into “To confirm whether the host factor for the *Zingipain-2-like* gene interacts with RNA silencing suppressing by the NSs gene of TZSV, the expression of the *Zingipain-2-like* gene performed by *NSs*, the silencing suppressor in plants, was detected by RT-qPCR assay, and found to be significantly decreased in the *NSs* gene-silenced plants compared to positive plants, compared with the CK, expression of the Zingipain-2-like gene was up-regulated in both positive and NSs-silenced plants infected by TZSV plants, but higher in the positive plant. The rationale behind these data could be that *Zingipain-2-like* gene expression was induced by NSs (Fig 3 C).”.

7) Line 245: “RNA silencing suppressor by NSs”, change to RNA silencing suppressor

NSs?

Answer: RNA silencing suppressor by NSs" has been changed change into "RNA silencing suppressing by the NSs gene".

8) Line 245: NSs made closely relationship with Zingipain-2-like…?

Answer: the words "interact with" have been changed into "made closely relationship with".

"make closely relationship with" is not proper English. Do you mean to say "expression of NSs is closely correlated to that of Zingipain-2-like gene?"

9) Legend to Fig. 3C: CK was not mentioned. Was CK a healthy plant?

Answer: Yes, "CK" a healthy plant, and has been inserted to Fig. 3C Legend.

10) Fig. 4: what were the organelles where the florescent signals were present?

Answer: The location of the co-expression of NSs-GFP with the Zingipain-2-like-RFP.

You did not answer the question. What organelles? Nuclei? Mitochondria? Chloroplasts? etc.

5. Discussion

1) Silencing of NSs seemed to have different impact on symptom expression on N. benthamiana and N. tabacum cv. K326. Why? – need a discussion.

Answer: Page 10, line 280-284: the section "TRV has a wide range of hosts, and there is a significant difference in sensitivity to TRV between species and cultivars [38, 39]. For instance, TRV sensitivity testing was carried out on 21 Gerbera cultivars, and only 5 cultivars showed photo bleached PDS silencing symptoms on newly developed leaves [40]." has been changed into " TRV has a wide range of hosts, with a significant difference in sensitivity to TRV between species and cultivars [38, 39]. For instance, TRV sensitivity testing was carried out on 21 gerbera cultivars, and the results revealed that only 5 cultivars showed photobleached PDS-silencing symptoms on newly developed leaves [40]." and transferred into the location was line 287 followed the word "species".

2) Line 259-262: "In the present study, the accumulation of the virus decreased and the symptom alleviated when the NSs gene was silenced, and we first revealed Zingipain-2-like gene might be associate with this function". – the data did not support this claim (see questions in Result, 5 and 6). The involvement of Zingipain-2-like gene in TZSV infection has to be verified in a well-defined study.

Answer: insert the sentence "The expression of the *Zingipain-2-like* gene was found to be significantly decreased in the *NSs* gene-silenced plants compared to positive plants,

compared with the CK, expression of the Zingipain-2-like gene was up-regulated in both positive and NSs-silenced plants infected by TZSV plants, but higher in the positive plant.” Before the word “and”.
