# Supplementary material for: Functional analysis of the nonstructural protein NSs of tomato zonate spot virus
Source: PLoS One. 2022 Jan 24;17(1):e0262194. doi: 10.1371/journal.pone.0262194 (PMC8786149; doi:10.1371/journal.pone.0262194)
Supplement: Attachment — Submitted filename: Response to reviews-2021.11.14.docx [file pone.0262194.s003.docx]

**Answer to reviews**

Review 1

1. Virus taxonomy and writing of scientific name and virus name. TZSV should belong to Tomato zonate spot orthotospovirus species, Orthotospovirus genus, Tospoviridae family, Bunyavirales order. Authors can visit the ICTV website for information. For abbreviation, a virus name is required. Therefore, the use of ‘tomato zonate spot virus (TZSV)’ is recommended.

**Answer:** I have changed “Tomato zonate spot orthotospovirus” into “tomato zonate spot virus” in the article.

2. The text needs to be completely corrected. Too many spelling and grammatical errors can be found in the text. In addition, the citation of some references seems inappropriate.

**Answer:** I have completely corrected the text and documented “changed point by point”; some references have been changed, including: Page 12, line 317-319: the reference “Wu XD, Wu XY, Li WB, Cheng XF. Molecular characterization of a divergent strain of calla lily chlorotic spot virus infecting celtuce (Lactuca sativa var. augustana) in China. Arch virol. 2018; 163(5): 1375-1378.” has been changed into “Zhu M, Jiang L, Bai BH, Zhao WY, Chen XJ, Li J, Yong Liu 2, Chen ZQ, Wang BT, Wang CL, Wu Q, Shen QH, Dinesh-Kumar SP, Tao XR. The intracellular immune receptor Sw-5b confers broad-spectrum resistance to Tospoviruses through recognition of a conserved 21-amino-acid viral effector epitope. Plant Cell. 2017; 29(9): 2214-2232.”Page 15, line 405-406: the reference “Kamoun, S. Groovy times: Filamentous pathogen effectors revealed. Curr. Opin. Plant Biol. 2007; 10: 358-365.” has been changed into “Gupta R, Min CW, Kim SW, Yoo JS, Moon AR, Shin AY, Kwon SY, Kim ST. A TMT-based quantitative proteome analysis to elucidate the TSWV induced signaling cascade in susceptible and resistant cultivars of *Solanum lycopersicum*. Plants (Basel) 2020; 9(3): 290.”

3. The legends of tables and figures have to be clearly described for readers to understand. Additionally, miscitation of tables and figures can be found. For example, the primers are listed in Table S1, but the citation is in Table 1 (page 4, line 93, and page 5, line 129); Table 2 is quoted at page 9 (line 229), there is actually no Table 2!

**Answer:** I have described the legends of tables and figures again in the “changed point by point file”; the citation is in Table 1 (page 4, line 93, and page 5, line 129) has been changed into “Table S1”; Table 2 is quoted at page 9 (line 229) has been changed into “Table 1”.

4. What is ‘PDS’, ‘TCS SP8’, ’PM’, etc.? When describing an acronym for the first time, the full name must be provided.

**Answer:** the full name was added when describing an acronym for the first time, for example, Phytoene dehydrogenase gene (*PDS*); insert the word “(PM)” followed the word membrane, confocal laser scanning microscope (CLSM); the acronym ‘TCS SP8’is the machine model.
5. The results, such as the quantification of NSs gene (standard curve and copy number, page 8, lines197-206) and the phenomenon and molecular evidence of PDS gene silencing in the tested plants (lines 207-223), should be illustrated in figures. Was PDS silencing suppressed by the TZSV NSs protein? It is not clear about the response to TZSV infection (or NSs protein) in the PDS-silenced tobacco plants! What is the point of this? In fact, I am confused about Fig. 2. I think the PDS silencing here is meaningless!

**Answer:** PDS silencing suppressed was not by the TZSV NSs protein, *PDS* as an indicator gene, the plant leaves will turn white when it was silenced that the leaves injected with pTRV-pTV00-PDS construct, the *NSs* gene might be silenced that the leaves injected the pTRV-pTV00-NSs construct.
6. Authors must explain why the Zingipain-2-like gene is investigated? It is not even described in the M & M section! Logically, preliminary research should be performed to reveal the possible role of Zingipain-2-like gene in orthotospoviral (or TZSV) infection.

**Answer:** the section “*NSs* gene sequence was amplified from total RNA isolated from tobacco infected by TZSV using reverse transcription PCR (RT-PCR) and the primers NSs-G-F/ NSs-G-R (Table 1). The NSs-N and NSs-C PCR Fragments were digested with Nco1and Spe1 and inserted into pCAMBIA-GFP using the same restriction sites to obtain pCAMBIA-NSs-N-GFP and pCAMBIA-NSs-C-GFP, respectively. The Golgi markers ST52-mCherry was amplified from total RNA isolated from tobacco Arabidopsis [21]” has been changed into “The sequences of *NSs* and *Zingipain-2-like* genes were amplified from the total RNA isolated from tobacco plants infected with TZSV using reverse transcription-PCR (RT-PCR) and the special primers (Table S1). The PCR fragments of *NSs* and *Zingipain-2-like* genes were digested with endonuclease and inserted into vector of pCAMBIA-GFP and pBI121-mCherry to obtain pCAMBIA-NSs -GFP and pBI121-Zingipain-2-like-mCherry, respectively. The Golgi marker ST52-mCherry was amplified from the total RNA isolated from both tobacco and Arabidopsis [21].”
7. The description of ‘replication’ of NSs gene or Zingipain-2-like gene is incorrect. It is should be ‘transcription’!

**Answer:** I have changed the word “replication” into “transcription” in the paper. For example: page 1, line 20: the word “replications” has been changed into “transcription”; Page 3, line 74: the word “replications” has been changed into “transcription”.
Reviewer #2: The manuscript (PONE-D-21-26999) describes the functional investigation of NSs gene of tomato zonate spot orthotospovirus. The authors showed that NSs protein (fused with GFP) localized in plasma membrane and Golgi bodies. They then constructed NSs-silenced tobacco plants by VIGS and then inoculated with tomato zonate spot orthotospovirus (TZSV). By monitoring the symptom expression and detection the NSs expression level, mild symptom was observed in N. benthamiana, but not in N. tabacum cv. K326. However, NSs was decreased by more than 90% in both plants. They also found that the expression of a host gene, Zingipain-2-like gene, seemed to be induced by the expression of NSs, and these two proteins co-localized in the cell. This was the first demonstration of the involvement of NSs in infection of TZSV. While the story is of interest, flaws need to be fixed before acceptance for publication.

1. Title: changed to “Functional analysis of the nonstructural protein NSs of tomato zonate spot orthotospovirus”

**Answer:** the title has been changed into “Functional analysis of the nonstructural protein NSs of tomato zonate spot virus”.
2. Abstract: needs to be rewritten after modification of the text.

**Answer:** the Abstract “Tomato zonate spot orthotospovirus (TZSV), a member of the genus *orthotospovirsus*, causing severe reductions of vegetables and ornamental crops in southwest China. The NSs protein is an RNA silencing suppressor in *orthotospovirsus*, such as TZSV, but the mechanism of NSs and its role in virus infection are poorly understood. Here, we showed that an NSs-GFP fusion protein transiently expressed on plasma membrane and the Golgi bodies in *Nicotiana benthamiana* plants. When TZSV *NSs* gene was silenced and infiltrated into *N. benthamiana* and *N*. *tabacum* cv. K326. RT-qPCR and Indirect enzyme-linked immunosorbent assay (ID-ELISA) analysis showed that *NSs* gene replications and protein expression were inhibited more than 90.00%, and the symptoms of the plants silenced alleviated. We also showed that the expression of *Zingipain-2-like* gene significant decreased when *NSs* genes was silenced, the NSs-GFP and Zingipain-2-like-mCheery fusion protein could co-localization expression. The results of this study provide new insight into the mechanism of silencing suppression by NSs and its effect on virus systemic infection, and support the theory of resistance breeding and preventing and controlling TZSV in the field.” has been changed into “Tomato zonate spot virus (TZSV), a member of the genus *orthotospovirsus*, causes severe damage to vegetables and ornamental crops in southwest China. The NSs protein is an RNA silencing suppressor in various *orthotospovirsus* like TZSV, but its mechanism and role in virus infection are poorly understood. Here, we observed that an NSs-GFP fusion protein was transiently expressed on the plasma membrane and Golgi bodies in *Nicotiana benthamiana* plants. The TZSV *NSs* gene was silenced and infiltrated into *N. benthamiana* and *N*. *tabacum* cv. K326. RT-qPCR and Indirect enzyme-linked immunosorbent assay (ID-ELISA) showed that the replications and the protein expression of the *NSs* gene were inhibited by more than 90.00%, and the symptoms on silenced plants were alleviated. We also found that the expression of the *Zingipain-2-like* gene significantly decreased when the *NSs* gene was silenced, resulting in co-localization of the NSs-GFP and the Zingipain-2-like-mCherry fusion protein. The findings of this study provide new insights into the mechanism of silencing suppression by NSs, as well as its effect on systemic virus infection, and also support the theory of disease resistance breeding and control and prevention of TZSV in the field.” .
3. Materials and Methods: materials and methods should be introduced in a logical manner, e.g., NSs gene amplification should go ahead of the construction of NSs gene silencing construct.

**Answer:** the sentence “The fragments of the to-be-silenced *NSs* gene were cloned in the pTV00 vectors.” has been transferred to the location before the sentence “The vectors were then transformed into *E. coli* DH5α competent cells.”.
1) pCAMBIA-GFP, where was it obtained?

**Answer:** The vector of pCAMBIA-GFP was obtained from our lab in the institute of biotechnology and germplasm resources, and insert this sentence before the sentence “The TZSV YN-Chili isolate was collected from infected field tomato plants in Yuanmou”.
2) Vector and construct are different. pTRV-pTV00-NSs is a construct, not a vector.

**Answer:** page 5, line 131: the words “pTRV-pTV00-NSs -vector” have been changed into “pTRV-pTV00-NSs -construct”;
3) Amount of inoculum used in leaf infiltration?

**Answer:** Inoculation was performed using a 1-mL syringe, and the inoculation solution of 1 mL was pressure injected into individual leave and per plant was injected 3 leaves.

4) Lines 141-143: Bleaching of the leaves of the PDS-positive control occurred at approximately 10-14 days post-inoculation, and TZSV was inoculated. — what did the authors mean?

**Answer:** Phytoene dehydrogenase gene (*PDS*) as an indicator gene, the plant leaves will turn white when it was silenced, so the leaves injected with PDS vector becoming bleaching at approximately 10–14 days, the leaves performed with pTRV-pTV00-NSs construct inoculated with TZSV.
5) Line1 164-165: The content of the genes was tested by ID-ELISA according to the antibody instructions to determine the antiviral activity of the VIGS. — not genes but proteins?

**Answer:** line 159-160: the sentence “The content of the genes was tested by ID-ELISA according to the antibody instructions to determine the antiviral activity of the VIGS.” has been changed into “The contents of the proteins were tested by ID-ELISA according to the instructions for antibodies to determine the antiviral activity of VIGS.”.
4. Results
1) Line 197: The copies of NSs gene was determined by RT-qPCR. — for what purpose?

**Answer:** The silencing efficiencies of the TZSV *NSs* gene were determined and the results indicate that the pTRV-pTV00-NSs VIGS vector was successfully constructed, TZSV infection was prevented due to the *NSs* gene might be associated with TZSV infection.
2) Line 212-217: the sentence needs to be reorganized, Fig.2A goes before Fig. 2B

**Answer:** Page 8, line 206-209: the sentence “TZSV was inoculated after 5 days, the positive control (inoculated with only TZSV) exhibited severe shrinkage symptoms (Fig 2B), and the leaves of *N. benthamiana* plants injected with the pTRV-pTV00-NSs vector before inoculation with TZSV began to display light shrinkage symptoms (Fig 2A),” has been changed into “After 5 days of inoculation with TZSV, the shrinkage appeared on the leaves of *N. benthamiana* plants injected with the pTRV-pTV00-NSs vector before inoculation with TZSV (Fig 2A). Severe leaf shrinkage also occurred in positive control plants (inoculated only with TZSV) (Fig 2B);”.
3) Line 212: TZSV was inoculated after 5 days,…--- compared with line 142 “10-14 days”?

**Answer:** two different assays. Firstly, Phytoene dehydrogenase gene (*PDS*) as an indicator gene, the plant leaves will turn white when it was silenced, so the leaves injected with PDS vector becoming bleaching at approximately 10–14 days; secondly, the leaves performed with pTRV-pTV00-NSs construct inoculated with TZSV. After 5 days of inoculation with TZSV, the shrinkage appeared on the leaves of *N. benthamiana* plants injected with the pTRV-pTV00-NSs vector before inoculation with TZSV (Fig 2A). Severe leaf shrinkage also occurred in positive control plants (inoculated only with TZSV) (Fig 2B).
4) Line 235: “the replication of Zingipain-2-like gene…”, changed to expression of Zingipain-2-like gene. Also the title of Fig. 3 should be changed accordingly.

**Answer:** Page 8, Line 235: the word “replication” has been changed into “expression”; Page 17, line 455: the title “The replication of *NSs* and *Zingipain-2-like* genes detected by RT-qPCR” has been changed into “RT-qPCR detected the transcription of *NSs* and *Zingipain-2-like* genes after *NSs* gene silenced”.
5) Line 234-238: Fig. 3C does not tell whether or not NSs interacts with Zingipain-2-like gene. The fact was that Zingipain-2-like gene expression was induced by NSs. Data in Fig. 3C was inaccurately explained in the text, compared with the CK, expression of the Zingipain-2-like gene was up-regulated in both normal and NSs-silenced plants infected by TZSV, but higher in the normal plant (positive plant). The rationale behind these data could be that the NSs in the positive plant was higher than in the NSs-silenced plant.

**Answer:** Page 8, line 227: insert the words “the regulation of” followed the word “in”. Page 8-9, line 228-232: the section “To confirm whether the host factor of *Zingipain-2-like* gene interact with RNA silencing suppressor by NSs of TZSV, the replication of *Zingipain-2-like* gene in plants was detected by RT-qPCR that *NSs* gene silenced. The results showed that the replication of *Zingipain-2-like* gene significantly decreased in *NSs* gene silenced plants compared to the positive plants (Fig 3 C).” has been changed into “To confirm whether the host factor for the *Zingipain-2-like* gene interacts with RNA silencing suppressing by the NSs gene of TZSV, the expression of the *Zingipain-2-like* gene performed by *NSs*, the silencing suppressor in plants, was detected by RT-qPCR assay, and found to be significantly decreased in the *NSs* gene-silenced plants compared to positive plants, compared with the CK, expression of the Zingipain-2-like gene was up-regulated in both positive and NSs-silenced plants infected by TZSV plants, but higher in the positive plant. The rationale behind these data could be that *Zingipain-2-like* gene expression was induced by NSs (Fig 3 C).”.

7) Line 245: “RNA silencing suppressor by NSs”, change to RNA silencing suppressor NSs?

**Answer:** RNA silencing suppressor by NSs” has been changed change into “RNA silencing suppressing by the NSs gene”.
8) Line 245: NSs made closely relationship with Zingipain-2-like…?

**Answer:** the words “interact with” have been changed into “made closely relationship with”.
9) Legend to Fig. 3C: CK was not mentioned. Was CK a healthy plant?

**Answer:** Yes, “CK” a healthy plant, and has been inserted to Fig. 3C Legend.
10) Fig. 4: what were the organelles where the florescent signals were present?
**Answer:** The location of the co-expression of NSs-GFP with the Zingipain-2-like-RFP.
5. Discussion
1) Silencing of NSs seemed to have different impact on symptom expression on N. benthamiana and N. tabacum cv. K326. Why? – need a discussion.

**Answer:** Page 10, line 280-284: the section “TRV has a wide range of hosts, and there is a significant difference in sensitivity to TRV between species and cultivars [38, 39]. For instance, TRV sensitivity testing was carried out on 21 Gerbera cultivars, and only 5 cultivars showed photo bleached PDS silencing symptoms on newly developed leaves [40].” has been changed into “ TRV has a wide range of hosts, with a significant difference in sensitivity to TRV between species and cultivars [38, 39]. For instance, TRV sensitivity testing was carried out on 21 gerbera cultivars, and the results revealed that only 5 cultivars showed photobleached PDS-silencing symptoms on newly developed leaves [40].” and transferred into the location was line 287 followed the word “species”.
2) Line 259-262: “In the present study, the accumulation of the virus decreased and the symptom alleviated when the NSs gene was silenced, and we first revealed Zingipain-2-like gene might be associate with this function”. – the data did not support this claim (see questions in Result, 5 and 6). The involvement of Zingipain-2-like gene in TZSV infection has to be verified in a well-defined study.

**Answer:** insert the sentence “The expression of the *Zingipain-2-like* gene was found to be significantly decreased in the *NSs* gene-silenced plants compared to positive plants, compared with the CK, expression of the Zingipain-2-like gene was up-regulated in both positive and NSs-silenced plants infected by TZSV plants, but higher in the positive plant.” Before the word “and”.
