# Supplementary material for: Functional analysis of the nonstructural protein NSs of tomato zonate spot virus
Source: PLoS One. 2022 Jan 24;17(1):e0262194. doi: 10.1371/journal.pone.0262194 (PMC8786149; doi:10.1371/journal.pone.0262194)
Supplement: Attachment — Submitted filename: Changes in list-2021.12.15.docx [file pone.0262194.s006.docx]

Changed point by point

1. Page 1, line 1: the title “Analysis function for a nonstructural NSs protein of tomato zonate spot orthotospovirus” has been changed into “Functional analysis of the nonstructural protein NSs of tomato zonate spot virus”.
2. Page 1, line 12: the word “orthotospovirus” has been changed into “virus”.
3. Page 1, line 13: the word “causing” has been changed into “causes”; the words “reductions of” has been changed into “damage to”.
4. Page 1, line 14: insert the word “various” followed the word “in”; delete the punctuation “,” followed the word “*orthotospovirsus*”; the words “such as” have been changed into “like”; the word “the” ha been changed into “its”.
5. Page 1, line 15: delete the words “of NSs” and “its”.
6. Page 1, line 16: the word “showed” has been changed into “observed”; insert the word “was” followed the word “protein”; insert the word “the” followed the word “on”; insert the word “(PM)” followed the word membrane.
7. Page 1, line 17: delete the word “the”; the word “When” has been changed into “The”.
8. Page 1, line 19: delete the word “analysis”; the words “*NSs* gene” have been changed into “the”.
9. Page 1, line 20: insert the word “the” before the word “protein”; insert the words “of the *NSs* gene” before the word “were”; insert the word “by” before the word “more”.; the word “replications” has been changed into “transcription”.
10. Page 1, line 21: the words “of the” have been changed into “on silenced”; the word “silenced” has been changed into “were”; the word “showed” has been changed into “found”.
11. Page 1, line 22-23: insert the word “the” before the word “*Zingipain-2-like*”; the word “significant” has been changed into “significantly”; insert the word “the” followed the word “when”; the word “genes” has been changed into “gene”; the sentence “the NSs-GFP and Zingipain-2-like-mCheery fusion protein could co-localization expression” has been changed into “resulting in co-localization of the NSs-GFP and the Zingipain-2-like-mCherry fusion protein”.
12. Page 1, line 24: the word “results” has been changed into “findings”; the word “insight” has been changed into “insights”.
13. Page 1, line 25: insert the punctuation “,” followed the word “NSs”; the word “and” has been changed into “as well as”; the word “virus” has been transferred to the location behind the word “systemic”; insert the word “also” before the word “support”; insert the word “disease” before the word “resistance”.
14. Page 1, line 26: the words “preventing and controlling” have been changed into “control and prevention of”.
15. Page 1, line 28: the word “orthotospovirus” has been changed into “virus”; insert the words “the dominant species” and the punctuation “,”.
16. Page 2, line 29-30: the word “*orthotospovirsus*” has been changed into “*orthotospovirus*”; delete the punctuation “,” in line 29; delete the words “is the dominant species of *orthospovirus*”; the word “and” has been changed into “,”.
17. Page 2, line 31: delete the word “and” before the word “Guangxi” and insert the punctuation “,” followed the word “Guangxi”; delete the word “in”.
18. Page 2, line 32: the words “Asian countries” have been changed into “Asia”.
19. Page 2, line 33: the words “ring” has been changed into “rings and”.
20. Page 2, line 34-36: the sentence “The host range of TZSV is very broad, and TZSV can infect more than 20 kinds of plant species belonging to 7 families, including economically important crops and weed species” has been changed into “TZSV has a very broad host range, infecting more than 20 types of economically important crops and weed species, which belong to 7 families”; insert the words “not only” before the word “led”.
21. Page 2, line 37: the words “plant species” have been changed into “plants”; the word “southwest” has been changed into “Southwest”; the words “and has” have been changed into “but also”.
22. Page 2, line 38: the word “of” has been changed into “for”.
23. Page 2, line 39: the word “consist” has been changed into “consists”; delete the word “virus”; the word “and” has been changed into “that”.
24. Page 2, line 40: insert the word “the” before the word “endoplasmic”; the word “of” has been changed into “in”, insert the number “1” before the number “5”.
25. Page 2, line 40: the word “*orthotospovirsus*” has been changed into “*orthotospovirus*”.
26. Page 2, line 42: insert the punctuation “,” before the word “and”; insert the word “The” before the letter “L” and insert the word “segment” followed the word “RNA”; insert the word “the” followed the word “encodes”.
27. Page 2, line 43: insert the word “The” before the letter “M” and insert the word “segment” followed the word “RNA”; the word “a” has been changed into “the”.
28. Page 2, line 44: insert the word “while” before the letter “S”; the word “a” has been changed into “the”.
29. Page 2, line 45: delete the parentheses and the letter “(N)”; insert the capital letter “N”, the punctuation “,” and the sentence “the NSs protein is a RNA-silencing suppressor” followed the word “protein”.
30. Page 2, line 46: the word “enveloping” has been changed into “the envelope formation of”; the word “genomic” has been changed into “genome”.
31. Page 2, line 47: delete the words “viruses in”.
32. Page 2, line 49: insert the punctuation “,” before the word “and”.
33. Page 2, line 50: the word “NSs” has been transferred to the location before the word “protein”; the word “a” has been changed into “the”; insert the words “that is” before the word “necessary”.
34. Page 2, line 51: the word “alters” has been changed into “can influence”; the word “reduces” has been changed into “suppress”.
35. Page 2, line 52: insert the word “the” before the word “enhanced”.
36. Page 2, line 53: the punctuation “,” has been changed into “.” before the word “NSs”; the word “also” has been transferred to the location followed the word “can”; delete the punctuation “,” before the word “and”.
37. Page 2, line 55: insert the word “the” before the word “NSs”; the word “in” has been changed into “protein of”; insert the word “the” before the word “function”; the word “on” has been changed into “of transmission of the virus”.
38. Page 2, line 56: delete the words “transmitted the virus”; the word “in” has been changed into “of”; delete the punctuation “,” and the words “is an activator of the” have been changed into “activates a”.
39. Page 2, line 57: the word “interacted” has been changed into “interact”; the word “VDAC” has been transferred to the location before the word “protein”.
40. Page 3, line 58: insert the punctuation “,” before the word “regulating”; insert the word “the” followed the word “of”.
41. Page 3, line 59: the word “suppressor” has been changed into “suppression”; the words “in TZSV” has been transferred to the location before the word “by”.
42. Page 3, line 60: the word “needed” has been changed into “need”.
43. Page 3, line 61: the word “determine” has been changed into “study”; the word “function” has been changed into “functions”.
44. Page 3, line 62: insert the word “the” followed the word “by”.
45. Page 3, line 64: insert the word “of” before the word “the”; the word “tomato” has been changed into “tomatoes”; insert the words “the expression of” before the word “increased”.
46. Page 3, line 65: the words “gene expression” have been changed into “genes”; the word “inhibit” has been changed into “prevent the”.
47. Page 3, line 67: the word “phytohormonessalicylic” has been changed into “phytohormone salicylic”; insert the word “the” before the word “infection”.
48. Page 3, line 69: insert the word “of” followed the word “silencing”; delete the word “the” followed the word “enhance”; delete the word “effect”.
49. Page 3, line 70-71: the word “defend against” have been changed into “combat the”; the sentence “Qiu et al. inserted the TSWV *N* gene into a TRV vector to further study the gene function” has been changed into “The *N* gene of TSWV was inserted into the TRV vector to further study the gene function”; the word “about” has been changed into “on”.
50. Page 3, line 72: the word “genes” has been changed into “gene” followed the word “*NSs*”.
51. Page 3, line 73: the word “onto” has been changed into “the”; insert the word “then” before the word “infiltrated”.
52. Page 3, line 74: the word “analysis” has been changed into “assays”; the word “replications” has been changed into “transcription”.
53. Page 3, line 75: the word “of” has been changed into “in”.
54. Page 3, line 76: insert the word “silenced” before the word “plants”; the word “silenced” has been changed into “were”; the word “showed” has been changed into “observed”; insert the word “the” followed the word “of”.
55. Page 3, line 77-79: the word “significant” has been changed into “significantly”; the word “genes” has been changed into “gene”; the sentence “The results also showed that the NSs-GFP and Zingipain-2-like-mCheery fusion protein could co-localization expression on plasma membrane in *Nicotiana benthamiana* plants.” has been changed into “Furthermore, the results showed the co-localization expression of NSs-GFP and Zingipain-2-like-mCherry fusion protein on the plasma membrane in *Nicotiana benthamiana* plants.”
56. Page 3, line 79-82: the sentence “Our findings wea firstly used TRV VIGS system to analysis TZSV gene function and have important new implications for mechanistic studies of silencing suppression by NSs and its effect on virus systemic infection.” has been changed into “This is the first report using the TRV VIGS system to analyze the functions of the TZSV gene, which have important new implications for mechanistic studies of the suppression of gene silencing by NSs and their effects on systemic infection by the virus.”.
57. Page 3, 4, line 85-91: the section “pTV00, pBINTRA, and pTV00-PDS vectors were provided by Professor Jianqiang Wu's laboratory, KIB CAS [20]. The TZSV YN-Chili isolate was collected from infected field tomato plants in Yuanmou, Yunnan Province, China, and was maintained in N. benthamiana [1]. N. tabacum cv. K326 and N. benthamiana were cultivated at the Yunnan Academy of Agricultural Science. Primers were designed by Primer 5 according to the sequences of the TZSV NSs genes published in the NCBI database (registration number: EF552433.1) (Table 1).” Has been changed into “The pTV00, pBINTRA, and pTV00-PDS vectors were provided by Professor Jianqiang Wu's laboratory at the Kunming Institute of Botany, Chinese Academy of Sciences (KIB CAS) [20]. The vector of pCAMBIA-GFP, pBI121-mCherry were obtained from our lab in the institute of biotechnology and germplasm resources. The TZSV YN-Chili isolate was collected from the infected tomato field in Yuanmou, Yunnan Province, China, and maintained on *N. benthamiana* [1]. *N. tabacum* cv. K326 and *N. benthamiana* were cultivated at the Yunnan Academy of Agricultural Science. Primers were designed using the Primer 5 Design Program based on the sequences of TZSV *NSs* gene and *Zingipain-2-like* gene published in the NCBI database (registration number: EF552433.1, LOC107763929) (Table S1).”
58. Page 4, line 93: the word “friction-based” has been changed into “frictional”.
59. Page 4, line 94: the number “6” has been changed into “six-”.
60. Page 4, line 95-96: insert the word “the” followed the word “in”; delete the parentheses “()”; insert the word “containing” before the number “137”; insert the word “and” before the number “3”; the words “after which” have been changed into “and there after”.
61. Page 4, line 97: insert the word “the” followed the word “after”.
62. Page 4, line 98-100: the sentence “Plants inoculated with PBS buﬀer were used as controls. After inoculation for 5 days, when symptoms appeared, five replicates were used for each sample.” has been changed into “Plant leaves inoculated with PBS buffer were used as controls, and five replicates were used for each sample. Five days after inoculation, symptoms appeared.”
63. Page 4, line 102: the word “of” has been changed into “provided in”.
64. Page 4, line 103: delete the word “the”; the word “A” has been changed into “The”.
65. Page 4, line 107: the words “primers, 2.5 µL of” have been changed into “and”; insert the word “was” before the word “added”.
66. Page 4, line 108: the words “so that” have been changed into “to the solution to obtain”; the word “was” has been changed into “of”; insert the words “denaturation at” followed the word “of”.
67. Page 4, line 110: the words “imaged by” have been changed into “analyzed using”.
68. Page 4, line 112: the sentence “The fragments of the *NSs* gene to be silenced were cloned into pTV00 vectors.” has been transferred to the location Page 5, line 119 and before the word “The”.
69. Page 4, line 114: insert the word “the” before the word “plant”.
70. Page 5, line 115: delete the word “the”; the word “with” has been changed into “using”.
71. Page 5, line 116: the word “amplification” has been changed into “amplified”; delete the word “the” before the word “pTV00”.
72. Page 5, line 118: insert the word “the” followed the word “into”.
73. Page 5, line 119-120: delete the words “*E. coli* DH5α”; insert the words “of *E. coli* strain DH5α” followed the word “cells”; the word “sent” has been changed into “transferred”.
74. Page 5, line 123-129: the section “*NSs* gene sequence was amplified from total RNA isolated from tobacco infected by TZSV using reverse transcription PCR (RT-PCR) and the primers NSs-G-F/ NSs-G-R (Table 1). The NSs-N and NSs-C PCR Fragments were digested with Nco1and Spe1 and inserted into pCAMBIA-GFP using the same restriction sites to obtain pCAMBIA-NSs-N-GFP and pCAMBIA-NSs-C-GFP, respectively. The Golgi markers ST52-mCherry was amplified from total RNA isolated from tobacco Arabidopsis [21].” has been changed into “The sequences of *NSs* and *Zingipain-2-like* genes were amplified from the total RNA isolated from tobacco plants infected with TZSV using reverse transcription-PCR (RT-PCR) and the special primers (Table S1). The PCR fragments of *NSs* and *Zingipain-2-like* genes were digested with endonuclease and inserted into vector of pCAMBIA-GFP and pBI121-mCherry to obtain pCAMBIA-NSs -GFP and pBI121-Zingipain-2-like-mCherry, respectively. The Golgi marker ST52-mCherry was amplified from the total RNA isolated from both tobacco and Arabidopsis [21].”.
75. Page 5, line 130: insert the word “the” before the word “VIGs”.
76. Page 5, line 131: the word “vector” has been changed into “constrcture”.
77. Page 5, line 132: insert the word “strain” before the word “GV3101”; insert the word “the” before the word “inoculation” insert the word “the” before the word “liquid”.
78. Page 5, line 133: the word “was” has been changed into “were”.
79. Page 5, line 134: the word “a” has been changed into “the”; insert the word “the” before the word “resuspended”.
80. Page 5, line 136-141: the section “Inoculations were performed using a 1 mL syringe, and the inoculation solution was pressure injected into 3 individual leaves per plant. Bleaching of the leaves of the PDS-positive control occurred at approximately 10-14 days post-inoculation, and TZSV was inoculated. Plants injected with pTRV-pTV00 and plants inoculated with TZSV alone served as positive controls. At approximately 5-10 days post-inoculation, samples were collected, and healthy plants were used as blank controls.” has been changed into “Inoculation was performed using a 1-mL syringe, and the inoculation solution was pressure injected into 3 individual leaves per plant. Inoculation of pTRV-pTV00-NSs construct was performed using a 1-mL syringe, and the inoculation solution of 1 mL was pressure injected into individual leave and per-plant was injected 3 leaves. Phytoene dehydrogenase gene (*PDS*) as an indicator gene, the plant leaves will turn white when it was silenced, so the leaves injected with pTRV-pTV00-PDS construct becoming bleached at approximately 10–14 days, the leaves performed with pTRV-pTV00-NSs construct inoculated with TZSV. Plants injected with pTRV-pTV00 and inoculated with TZSV alone served as positive controls. At approximately 5–10 days post-inoculation, samples were collected and healthy plants were used as blank controls. ”
81. Page 5, line 142: delete the word “assays”
82. Page 5, line 143: the word “Leaf” has been changed into “The leaf”; the word “were” has been changed into “was”; insert the words “areas of the” before the word “agroinfiltrated”; delete the words “leaf area of”; the letter “N” has been changed into Italic.
83. Page 6, line 144: the word “mounted” has been changed into “placed”; the word “Confocal” has been changed into “The confocal”.
84. Page 6, line 145-146: the word “an” has been changed into “the”; the sentence “GFP was excited with 488 nm wavelength and emissions at 497-520 nm captured” has been changed into “GFP was excited at a wavelength of 488 nm, and emissions were captured at 497–520 nm”.
85. Page 6, line 146-147: the sentence “mCherry were excited with 561 nm wavelength and emissions at 585–615 nm captured.” has been changed into “Moreover, mCherry was excited at a wavelength of 561 nm and emissions were captured at 585–615 nm.”.
86. Page 6, line 148: the word “a” has been changed into “the”.
87. Page 6, line 150: insert the word “the” followed the word “into”; the word “vectors” has been changed into “vector”.
88. Page 6, line 151: delete the words “with a”; the word “continuous” has been changed into “with a”; the words “at” has been changed into “with”.
89. Page 6, line 152: the word “concentration differences” has been changed into “dilution series”; the words “which were” have been changed into “and then”.
90. Page 6, line 153: delete the word “following”.
91. Page 6, line 154: the punctuation “:” has been changed into “including”; the words “primers, 2.5 µL of” have been changed into “and”.
92. Page 6, line 156: insert the words “initial denaturation step at” before the number “95”.
93. Page 6, line 159-160: the sentence “The content of the genes was tested by ID-ELISA according to the antibody instructions to determine the antiviral activity of the VIGS.” has been changed into “The contents of the proteins were tested by ID-ELISA according to the instructions for antibodies to determine the antiviral activity of VIGS.”.
94. Page 6, line 161: the word “with” has been changed into “using”; insert the words “and diluted 1: 3” before the word “in”; delete the words “1/3 dilution with”.
95. Page 6, line 162: the word “to” has been changed into “into”.
96. Page 6, line 163: the word “conjugate” has been changed into “a conjugation”.
97. Page 6, line 164: delete the punctuation “.”; the word “Afterward” has been changed into “and afterward”; delete the words “AP-conjugated”; insert the words “-AP conjugated” followed the word.
98. Page 6, line 166: insert the word “obtain” before the word “a”.
99. Page 6, line 167: insert the words “was determined” before the word “at”; delete the words “was measured”; the word “a” has been changed into “the”.
100. Page 6, line 169: delete the sentence “PBS buffer was used as a blank control,”.
101. Page 6, line 170: insert the sentence “The PBS buffer was used as a blank control.” followed the word “control”.
102. Page 7, line 172: the word “is” has been changed into “was”.
103. Page 7, line 174-175: the word “always” has been changed into “was”; the word “analysis” has been changed into “analyze the”; the letter “N” has been changed into italic; the words “an ideal” has been changed into “a model”; delete the words “in which”.
104. Page 7, line 176: the word “target” has been changed into “targeting”; the words “we first transiently expressed” has been changed into “the”.
105. Page 7, line 177: insert the words “was first transiently expressed” before the word “in”.
106. Page 7, line 178: the letter “N” has been changed into italic.
107. Page 7, line 178-179: the sentence “We detected NSs-GFP in a structure that was plasma membrane by CLSM” has been changed into “NSs-GFP was then detected to be associated with the plasma membrane structures by CLSM”.
108. Page 7, line 180: insert the word “are” before the word “colocalized”.
109. Page 7, line 181: insert the word “the” before the word “marker”.
110. Page 7, line 181-183: the sentence “After agroinfiltration of N. *benthamiana* 48 h, we found that the NSs-GFP bodies colocalized with the Golgi stacks (Fig 1 D-F), suggesting that the NSs-GFP protein targets to the Golgi apparatus.” has been changed into “At 48 h after agroinfiltration on *N.* *benthamiana*, we found that the NSs-GFP bodies are colocalized with the Golgi stacks (Fig 1 D-F), suggesting that the NSs-GFP protein was targeted to the Golgi apparatus.”.
111. Page 7, line 185: the word “is” has been changed into “was”; insert the word “the” before the word “interactions”.
112. Page 7, line 186: the word “Specific” has been changed into “specific”.
113. Page 7, line 187: insert the word “recognition” before the word “sites”.
114. Page 7, line 188: insert the punctuation “,” before the word “and”; the word “a” has been changed into “into the”.
115. Page 7, line 191: insert the word “the” before the word “*NS*s”; the words “was determined” have been changed into “were analyzed”.
116. Page 7, line 192: the word “a” has been changed into “the”.
117. Page 7, line 193: the word “concentrations” has been changed into “concentration”; insert the word “the” before the word “OD”; the punctuation “,” has been changed into “were”.
118. Page 7, line 194: insert the punctuation “,” and the word “respectively” followed the number “1.83”.
119. Page 7, line 194-195: the sentence “Gradient dilutions (10^-1^ to 10^-6^) of plasmid DNA was used as templates.” has been changed into “The plasmid DNA with gradient dilutions of 10^–1^ to 10^–6^ was used as a template.”; the word “of” has been changed into “for”; the word “genes” has been changed into “gene”.
120. Page 7, line 196: the word “was” has been changed into “were”; the word “software” has been transferred to the location before the word “system”.
121. Page 7, line 197: the word “equations” has been changed into “equation”; insert the word “and” followed the punctuation “,”.
122. Page 7, line 197-198: the words “efficiencies were 90.90%” have been changed into “efficiency”; the words “coefficients was 0.998” have been changed into “coefficient were 90.90% and 0.998, respectively”.
123. Page 7, line 199: the words “could be used” have been transferred to the location before the word “as”; the word “measure” has been changed into “determine”.
124. Page 8, line 201: the words “indicate whether” have been changed into “ensure”; insert the words “success of” before the word “silencing”; delete the word “was” followed the word “silencing”.
125. Page 8, line 202: delete the word “successful”; the sentence “Without this gene in the plant, the *N. benthamiana* leaves were bleached.” has been changed into “The leaves of *N. benthamiana* without this gene were bleached.”.
126. Page 8, line 203: the word “the” has been transferred to location behind the word “of”.
127. Page 8, line 204-205: the word “this” has been changed into “the fact that these”; the word “belonging” has been changed into “belong”; the sentence “TZSV was inoculated into both plants when the leaves occurred white in the veins of *N. benthamiana* leaves.” has been changed into “TZSV was inoculated on the two species tobaccos that the plants were injected with pTRV-pTV00-NSs construct and positive control plants (inoculated only with TZSV), respectively, when the leaves in the veins of *N. benthamiana* turned white injecting with pTRV-pTV00-PDS construct.”.
128. Page 8, line 206-209: the sentence “TZSV was inoculated after 5 days, the positive control (inoculated with only TZSV) exhibited severe shrinkage symptoms (Fig 2B), and the leaves of *N. benthamiana* plants injected with the pTRV-pTV00-NSs vector before inoculation with TZSV began to display light shrinkage symptoms (Fig 2A),” has been changed into “After 5 days of inoculation with TZSV, the shrinkage appeared on the leaves of *N. benthamiana* plants injected with the pTRV-pTV00-NSs vector before inoculation with TZSV (Fig 2A). Severe leaf shrinkage also occurred in positive control plants (inoculated only with TZSV) (Fig 2B);”.
129. Page 8, line 209-210: the words “were not different” have been transferred to the location behind the word “plants”; the plant “between” has been changed into “in”.
130. Page 8, line 211-213: the sentence “the expression of the *NSs* gene was measured by RT-qPCR. RT-qPCR analysis showed that the expression of the *NSs* gene significantly decreased compared to that of the positive control (inoculated with only TZSV) in both hosts,” has been changed into “its transcription level was measured by RT-qPCR and found to be significantly decreased compared to that of the positive control (inoculated only with TZSV) in both hosts.”; delete the word “and”.
131. Page 8, line 214: the word “efficiency” has been changed into “efficiencies”; the word “was” has been changed into “were”; delete the punctuation “,”.
132. Page 8, line 215: the words “Taken together, the” have been changed into “The”; the word “indicating” has been changed into “indicate”.
133. Page 8, line 216: the word “successful” has been changed into “successfully”; insert the punctuation “,” before the word “and”; insert the word “the” before the word “*NSs*”; insert the word “might” followed the word “might”.
134. Page 8, line 218: insert the word “the” followed the word “of”.
135. Page 8, line 219-222: the section “The NSs protein contents was measured by ID-ELISA at 3 d, 5 d, 7d, and 9 d post inoculation with TZSV. The content of NSs protein in *N. benthamiana* leaves and *N. tabacum* cv. K326 treated with the pTRV-pTV00-NSs vector and inoculated with TZSV significantly decreased compared to that in the positive plants at 7d and 9 d (Table 2).” has been changed into “The NSs protein levels were measured by ID-ELISA at 3, 5, 7, and 9 days post-inoculation with TZSV, and found to be significantly decreased in leaves of *N. benthamiana* and *N. tabacum* cv. K326 plants treated with the pTRV-pTV00-NSs construct and inoculated with TZSV compared to those in positive plants at 7 and 9 days (Table 1).”.
136. Page 8, line 223: insert the word “the” followed the word “of” and insert the word “both” followed the word “in”.
137. Page 8, line 225: the sentence “NSs gene silencing depending on zingipain-2-like gene” has been changed into “The dependence of NSs gene silencing on the zingipain-2-like gene”.
138. Page 8, line 226: insert the word “the” before the word “*Zingipain-2-like*”; insert the word “a” followed the word “was”.
139. Page 8, line 227: insert the words “the regulation of” followed the word “in”.
140. Page 8-9, line 228-232: the section “To confirm whether the host factor of *Zingipain-2-like* gene interact with RNA silencing suppressor by NSs of TZSV, the replication of *Zingipain-2-like* gene in plants was detected by RT-qPCR that *NSs* gene silenced. The results showed that the replication of *Zingipain-2-like* gene significantly decreased in *NSs* gene silenced plants compared to the positive plants (Fig 3 C).” has been changed into “To confirm whether the host factor for the *Zingipain-2-like* gene interacts with RNA silencing suppressing by the NSs gene of TZSV, the expression of the *Zingipain-2-like* gene performed by *NSs*, the silencing suppressor in plants, was detected by RT-qPCR assay, and found to be significantly decreased in the *NSs* gene-silenced plants compared to positive plants, compared with the CK, expression of the Zingipain-2-like gene was up-regulated in both positive and NSs-silenced plants infected by TZSV plants, but higher in the positive plant. The rationale behind these data could be that *Zingipain-2-like* gene expression was induced by NSs (Fig 3 C).”.
141. Page 9, line 233: the word “identify” has been changed into “investigate”; insert the word “the” before the word “Zingipain-2-like”; insert the word “were” followed the word “protein”; insert the words “to identify” before the word “the”.
142. Page 9, line 234: insert the word “the” before the word “recombinant”.
143. Page 9, line 236: the letter “N” has been changed into italic. The words “Co-expression” have been changed into “The co-expression”.
144. Page 9, line 237: insert the words “the co-localization of” before the word “NSs”; the words “co-localized, and localization to” have been changed into “at”.
145. Page 9, line 238: the word “CLSM” has been changed into “the confocal laser scanning microscope (CLSM)”; the word “clarify” has been changed into “clarified”.
146. Page 9, line 238-240: the sentence “Our results clarify that RNA silencing suppressor by NSs made closely relationship with Zingipain-2-like when TZSV infected the plants.” has been changed into “Our results clarified that expression of *NSs* is closely correlated to that of Zingipain-2-like when TZSV infected the plants.”
147. Page 9, line 242: the words “in plants” has been transferred to location before the word “was”
148. Page 9, line 243: the punctuation “,” has been changed into “.”, the letter “a” has been changed into capital; the word “was” has been changed into “were”.
149. Page 9, line 244: the word “when” has been changed into “with the”; delete the word “which”.
150. Page 9, line 245: delete the punctuation “,”.
151. Page 9, line 246: the words “suppressing local” have been changed into “suppress short”; the word “virus” has been changed into “viral”.
152. Page 9, line 248: insert the punctuation “,” and the words “a regulator” followed the word “*MYC2*”; insert the punctuation “,” followed the word “signaling”.
153. Page 9, line 249: the word “attenuates” has been changed into “attenuate”; the word “increases” has been changed into “increasing”.
154. Page 9, line 250: the word “attraction” has been changed into “attractiveness”; insert the words “the plants to” followed the word “of”; insert the punctuation “,” followed the word “thrips”; the words “to transmit” has been changed into “and thus transmitting”.
155. Page 9, line 250-251: the sentence “So far, the ability of TSWV NSs to counteract the RNA silencing defense mechanism in plants was illuminated,” has been changed into “So far, the ability of NSs of TSWV to counteract defense mechanisms mediated by RNA silencing in plants has been demonstrated;”.
156. Page 9, line 252: insert the words “NSs in” followed the word “by”; delete the word “NSs”.
157. Page 9, line 253: the word “have” has been changed into “are”; the words “been clearly” have been changed into “yet clear”.
158. Page 9, line 254: the words “the symptom” have been changed into “symptoms were”; the punctuation “,” has been changed into “.”; insert the sentence “The expression of the *Zingipain-2-like* gene was found to be significantly decreased in the *NSs* gene-silenced plants compared to positive plants, compared with the CK, expression of the Zingipain-2-like gene was up-regulated in both positive and NSs-silenced plants infected by TZSV plants, but higher in the positive plant.” Before the word “and”. the words “and we” have been changed into “We”.
159. Page 9, line 255: insert the words “that the” followed the word “revealed”; the word “associate” has been changed into “associated”.
160. Page 9, line 256: delete the word “plant’s”; the word “contained” has been changed into “of plants has”; the punctuation “:” has been changed into “,”; insert the word “including” before the word “microbe”.
161. Page 9, line 257: insert the word “(MAMP)” followed the word “associated”.
162. Page 9, line 258: insert the word “the” followed the word “by”.
163. Page 9, line 259: the word “of” has been changed into “in”.
164. Page 10, line 260: insert the word “the” before the word “pathogen”; insert the word “that” followed the word “proteins”.
165. Page 10, line 261: delete the words “were reported”; the words “such as TSWV” have been changed into “like the”; insert the words “of TSWV that” followed the word “protein”.
166. Page10, line 262: the words “and PTI was inhibited” have been changed into “, have been reported”.
167. Page 10, line 263: insert the word “were” followed the word “like”; the word “to” has been changed into “at”; the word “and” has been changed into “; the expression of the”.
168. Page 10, line 264: delete the word “the”.
169. Page 10, line 265: insert the word “and” before the word “Zingipain”; the word “take” has been changed into “took”; insert the words “the regulation of” before the word “hypersensitive”; the words “it suggested” have been changed into “suggesting”.
170. Page 10, line 266: the words “to activate” have been changed into “with the activation of”.
171. Page 10, line 267: the letter “N” has been changed into italic; the word “system” has been changed into “plant”; insert the punctuation “,” before the word “and”; the words “the first time found” have been changed into “the ability of the”.
172. Page 10, line 268: the word “could” has been changed into “to”; delete the word “into”; insert the words “was observed for the first time” followed the word “cells”; the word “viral” has been changed into “the virus”.
173. Page 10, line 270-271: the word “for” has been changed into “of”; the word “in” has been changed into “belonging to”; the punctuation “,” has been changed into “.”; the word “Why NSs protein facilitate” has been changed into “However, the reason for the facilitation of”; delete the word “an”; insert the words “particles by the NSs protein” followed the word “spherical”.
174. Page 10, line 270-272: delete the words “in the future”.
175. Page 10, line 273: the word “to” has been changed into “for the”.
176. Page 10, line 274: the words “analysis the function” have been changed into “of their functions”; insert the punctuation “,” followed the word “qPCR”; the word “and” has been changed into “as well as”; insert the word “disease” followed the word “plant”.
177. Page 10, line 275: the word “to” has been transferred to the location followed the word “up”.
178. Page 10, line 276: insert the word “gene” before the word “silencing”; the word “measured” has been changed into “verified”.
179. Page 10, line 277: the word “when” has been changed into “at”; insert the word “the” before the word “disease”; delete the words “appears on the plants”.
180. Page 10, line 278: the word “has” has been changed into “had”; the word “of” has been changed into “in”.
181. Page 10, line 279: the punctuation “;” has been changed into “,”; insert the word “and” before the word “thus”; insert the word “at” followed the word “silenced”.
182. Page 10, line 280: insert the word “levels” followed the word “high”.
183. Page 10, line 280-284: the section “TRV has a wide range of hosts, and there is a significant difference in sensitivity to TRV between species and cultivars [38, 39]. For instance, TRV sensitivity testing was carried out on 21 Gerbera cultivars, and only 5 cultivars showed photo bleached PDS silencing symptoms on newly developed leaves [40].” has been changed into “ TRV has a wide range of hosts, with a significant difference in sensitivity to TRV between species and cultivars [38, 39]. For instance, TRV sensitivity testing was carried out on 21 gerbera cultivars, and the results revealed that only 5 cultivars showed photobleached PDS-silencing symptoms on newly developed leaves [40].” and transferred into the location was line 287 followed the word “species”.
184. Page 10, line 285: delete the word “the”; insert the words “the fact that” followed the word “despite”.
185. Page 10, line 286: the word “belonging” has been changed into “belong”; the word “different” has been changed into “differing”.
186. Page 11, line 288: the word “research” has been changed into “studies”; the words “to determine” have been changed into “the analysis of”.
187. Page 11, line 289: delete the words “presented here”; insert the word and the punctuation “NSs,” followed the word “that”.
188. Page 11, line 290: the words “NSs of” have been changed into “in”; insert the word “was” before the word “localized”; insert the word “also” before the word “might”.
189. Page 11, line 291: the words “taking advantage of” have been changed into “using”.
190. Page 11, line 292: the word “methods” hasbeen changed into “prediction”.

Page 11, line 297-298: insert the content “Supporting information S1Table. Special primers. The primers were used to construct the VIGS, fluorescence labeling, and RT-qPCR vectors. S1 File. Amplication of the *NSs* gene sequence for VIGs.”.

191. Page 11, line 308-310: the section “**Funding** This study was supported by National Natural Science Foundation of China (No. U1802235, 31960531), Yunnan Fundamental Research Projects (202101AT070269). Has been changed into “**Data Availability Statement** All relevant data are within the manuscript and its Supporting information files. **Funding**This study was supported by National Natural Science Foundation of China (No. U1802235, 31960531), Yunnan Fundamental Research Projects (202101AT070269). The funders had no role in study design, data collection and analysis, decision to publish, or preparation of the manuscript. **Competing interests** The authors have declared that no competing interests exist.”
192. Page 12, line 317-319: the reference “Wu XD, Wu XY, Li WB, Cheng XF. Molecular characterization of a divergent strain of calla lily chlorotic spot virus infecting celtuce (Lactuca sativa var. augustana) in China. Arch virol. 2018; 163(5): 1375-1378.” has been changed into “Zhu M, Jiang L, Bai BH, Zhao WY, Chen XJ, Li J, Yong Liu 2, Chen ZQ, Wang BT, Wang CL, Wu Q, Shen QH, Dinesh-Kumar SP, Tao XR. The intracellular immune receptor Sw-5b confers broad-spectrum resistance to Tospoviruses through recognition of a conserved 21-amino-acid viral effector epitope. Plant Cell. 2017; 29(9): 2214-2232.”
193. Page 13, line 369: insert the words (in Chinese) at the end of the sentence.
194. Page 14, line 385: the words “Biochemical & Biophysical Research Communications” have been changed into “Biochem Biophys Res Commun”.
195. Page 15, line 404: delete the punctuation “.” followed the word “Mol”
196. Page 15, line 405-406: the reference “Kamoun, S. Groovy times: Filamentous pathogen effectors revealed. Curr. Opin. Plant Biol. 2007; 10: 358-365.” has been changed into “Gupta R, Min CW, Kim SW, Yoo JS, Moon AR, Shin AY, Kwon SY, Kim ST. A TMT-based quantitative proteome analysis to elucidate the TSWV induced signaling cascade in susceptible and resistant cultivars of *Solanum lycopersicum*. Plants (Basel) 2020; 9(3): 290.”
197. Page 15, line 416: insert the punctuation “.” followed the word “Cells”.
198. Page 16, line 435: the words “after VIGS by” have been changed into “with different treatment in both host by using”.
199. Page 16, line 437: delete the word “means”; the words “VIGS vector” have been changed into “construct”; insert the word “with” before the word “TZSV”.
200. Page 16, line 438: delete the word “VIGS”.
201. Page 16, line 442: the title “TZSV NSs is localized with the PM and Golgi bodies” has been changed into “The NSs protein in TZSV is localized at the PM and Golgi bodies”.
202. Page 16, line 445: the word “Colocalization” has been changed into “co-localization”.
203. Page 17, line 450: the title “Fig 2 The symptoms on N. *benthamiana* after NSs gene silenced” has been changed into “The symptom of TZSV infection on *N*. *benthamiana*”.
204. Page 17, line 452-453: the content “A: the plant of infiltrating pTRV-PTV00-NSs vector firstly and inoculated with TZSV; B: posivite control of the plant inoculated with TZSV only; C: negative control” has been changed into “A: infiltrating pTRV-PTV00-NSs construct in the leaves and inoculated with TZSV; B: posivite control (inoculated with TZSV only); C: negative control (the healthy plant)”.
205. Page 17, line 455: the title “The replication of *NSs* and *Zingipain-2-like* genes detected by RT-qPCR” has been changed into “RT-qPCR detected the transcription of *NSs* and *Zingipain-2-like* genes after *NSs* gene silenced”.
206. Page 17-18, line 457-463: the content “A: detection of *NSs* gene expression after VIGS and inoculated TZSV at 5d in *Nicotiana Benthamiana* leaves; B: detection of *NS* gene expression after VIGS and inoculated TZSV at 5d in *N. tabacum* cv. K326 leaves; C: detection of *Zingipain-2-like* gene expression after VIGS and inoculated TZSV at 5d in *Nicotiana Benthamiana* leaves; VIGS-NSs: infiltrated pTRV-pTV00-NSs VIGS vector and inoculated TZSV; pTV00-TZSV: infiltrated pTRV-pTV00 VIGS vector and inoculated TZSV; TZSV: positive control and only inoculated with TZSV. All values are means ± SE. *means differences are significantly different at P ≤ 0.05.” has been changed into “A: the expression of *NSs* gene is detected with different treatment in *Nicotiana Benthamiana* leaves; B: the expression of *NSs* gene is detected with different treatment in *N. tabacum* cv. K326 leaves; C: the expression of *Zingipain-2-like* gene is detected in *Nicotiana Benthamiana* leaves; VIGS-NSs: infiltrated pTRV-pTV00-NSs construct and inoculated with TZSV; pTV00-TZSV: infiltrated pTRV-pTV00 vector and inoculated with TZSV; TZSV: positive control and only inoculated with TZSV; CK: negative control (the healthy plant). All values are means ± SE. *means differences are significantly different at P ≤ 0.05. ”
207. Page 18, line 465: the title “Fig 4 TZSV NSs is localized with Zingipain-2-like protein” has been changed into “Fig 4 The NSs protein in TZSV is co-localized with the Zingipain-2-like protein”.
208. Page 18, line 467: the word “Colocalization” has been changed into “co-localization”
